# A novel calcimimetic agent, evocalcet (MT-4580/KHK7580), suppresses the parathyroid cell function with little effect on the gastrointestinal tract or CYP isozymes *in vivo* and *in vitro*

**DOI:** 10.1371/journal.pone.0195316

**Published:** 2018-04-03

**Authors:** Takehisa Kawata, Shin Tokunaga, Miki Murai, Nami Masuda, Waka Haruyama, Youji Shoukei, Yutaka Hisada, Tetsuya Yanagida, Hiroshi Miyazaki, Michihito Wada, Tadao Akizawa, Masafumi Fukagawa

**Affiliations:** 1 Nephrology Research Laboratories, Nephrology R&D Unit, R&D Division, Kyowa Hakko Kirin Co., Ltd., Shizuoka, Japan; 2 Research Core Function Laboratories, Research Functions Unit, R&D Division, Kyowa Hakko Kirin Co., Ltd., Shizuoka, Japan; 3 Takasaki Plant, Production Division, Kyowa Hakko Kirin Co., Ltd., Gunma, Japan; 4 Research Unit, Innovative Research Division, Mitsubishi Tanabe Pharma Corporation., Kanagawa, Japan; 5 Medical Affairs Department, Kyowa Hakko Kirin Co., Ltd., Tokyo, Japan; 6 Division of Nephrology, Department of Medicine, Showa University School of Medicine, Tokyo, Japan; 7 Division of Nephrology, Endocrinology and Metabolism, Tokai University School of Medicine, Kanagawa, Japan; Nanjing Medical University, CHINA

## Abstract

Cinacalcet hydrochloride (cinacalcet), an oral calcimimetic agent has been widely used for the management of secondary hyperparathyroidism (SHPT) in chronic kidney disease (CKD). In sharp contrast to vitamin D receptor activators, cinacalcet suppresses SHPT without inducing hypercalcemia or hyperphosphatemia. Nevertheless, some patients remain refractory to SHPT with this agent, as the dose cannot be sufficiently increased due to gastrointestinal symptoms. In order to resolve this issue, we have developed a newly synthesized calcimimetic agent, evocalcet (MT-4580/KHK7580). In a rat model of CKD induced by 5/6 nephrectomy, oral administration of evocalcet efficiently suppressed the secretion of parathyroid hormone (PTH). With regard to the gastro-intestinal effects, cinacalcet induced a significant delay in gastric emptying in rats, while evocalcet did no marked effects on it. Evocalcet also demonstrated the less induction of emesis compared to cinacalcet in common marmosets. The pharmacological effects of evocalcet were observed at lower doses because of its higher bioavailability than cinacalcet, which may have contributed to the reduced GI tract symptoms. In addition, evocalcet showed no substantial direct inhibition of any CYP isozymes in *in vitro* liver microsome assay, suggesting a better profile in drug interactions than cinacalcet that inhibits cytochrome P450 (CYP) 2D6. These findings suggest that evocalcet can be a better alternative to cinacalcet, an oral calcimimetic agent, with a wider safety margin.

## Introduction

Secondary hyperparathyroidism (SHPT), characterized by the elevation of serum parathyroid hormone (PTH) levels, is a common disorder in patients with chronic kidney disease (CKD), especially those on renal replacement therapy [1]. As CKD progresses, an extreme increase in the serum PTH levels results in high-turnover bone disease and increases the serum calcium and phosphate levels. Such abnormal mineral metabolism often results in vascular calcification, fracture, and an increased risk of all-cause and cardiovascular mortality [2–5].

Cinacalcet hydrochloride (cinacalcet), a calcimimetic agent that allosterically activates the calcium receptor (CaR) on parathyroid gland cells and suppresses PTH secretion [6,7]. Cinacalcet has been widely used to manage SHPT in dialysis patients [8–13], and is associated with a reduced risk of cardiovascular calcification, hospitalization and heart failure [14–16]. As a result, cinacalcet has helped to drastically reduce the number of parathyroidectomy (PTx) surgeries [17].

However, cinacalcet treatment is occasionally associated with gastrointestinal (GI) symptoms, including nausea and vomiting [18]. Such GI intolerability limits the dose of cinacalcet and may result in poor compliance or discontinuation [19,20]. Given reports of cinacalcet inhibiting gastric emptying in hemodialysis patients [21], delayed gastric emptying seems to contribute to GI events caused by cinacalcet treatment. We therefore hypothesized that abnormal GI motility might be a mechanism underlying GI events and a good marker of side effects in the GI tract.

Furthermore, cinacalcet has an inhibitory effect on cytochrome P450 (CYP) 2D6, which has raised concerns on interactions with a number of drugs [22,23]. Considering the issues that are associated cinacalcet, there is an unmet need for novel calcimimetic agents with an improved profile or fewer side effects.

Evocalcet (MT-4580/KHK7580) is a novel oral calcimimetic compound that was developed by screening for the ability to activate CaR *in vitro* and by evaluating the emetic effect *in vivo*. The purpose of this study was to characterize the pharmacological profiles of evocalcet.

## Materials and methods

### Evocalcet

**Fig 1:**
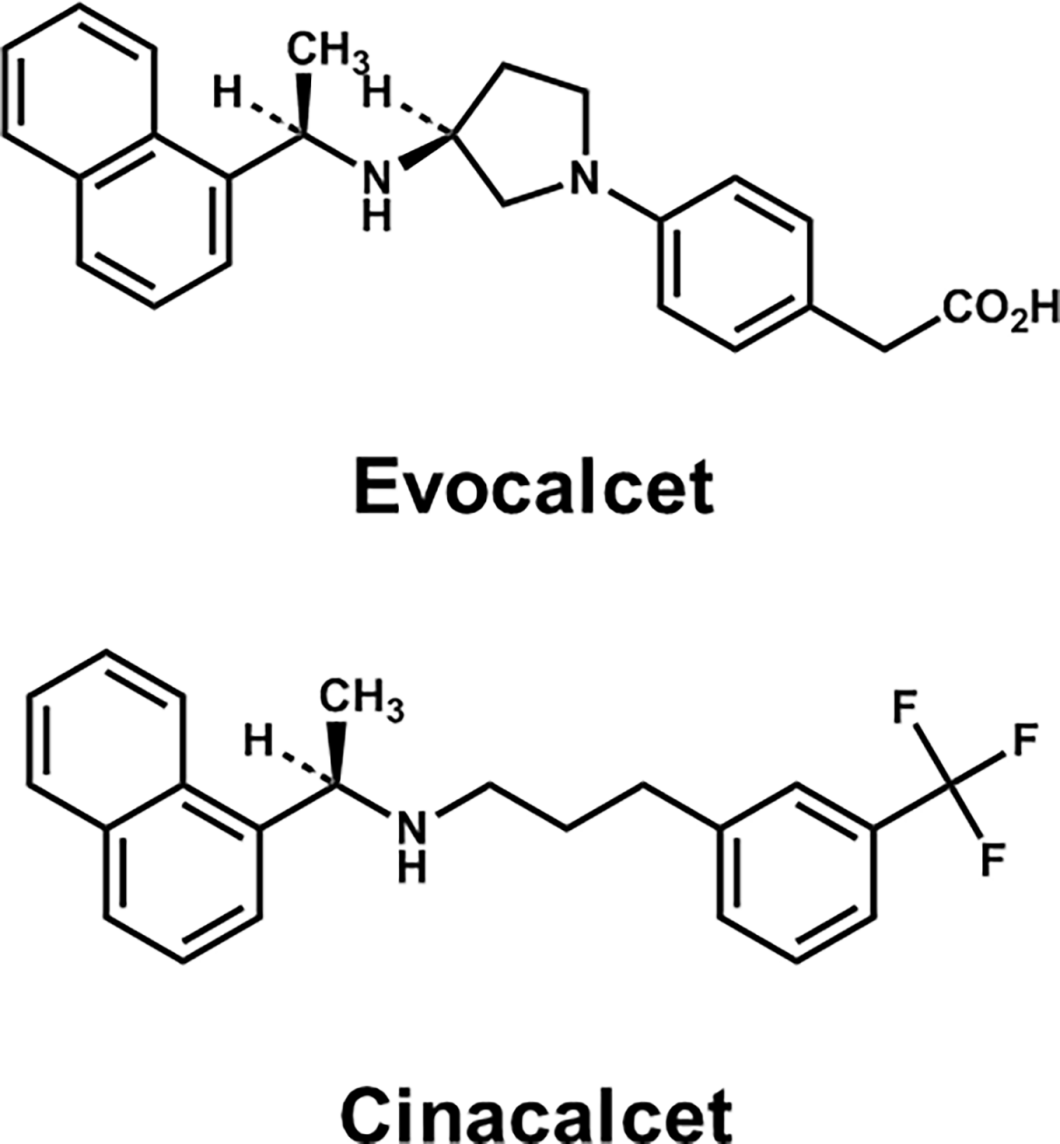
The chemical structure of evocalcet and cinacalcet.

Evocalcet was synthesized at Mitsubishi Tanabe Pharma Corporation (Osaka, Japan). Evocalcet is a compound containing naphthylethylamine skeletons, just like cinacalcet (Fig 1). Many calcimimetics reported in the literature are phenylalkylamine chemotype analogs, such as NPS R-568, and bind to the transmembrane domain of CaR [24].

### Agonistic activity studies with HEK293 cells stably expressing human CaR

HEK 293 cell is obtained from Summit Pharmaceuticals International Corporation (ATCC^®^ Number: CRL-1573^TM)^. The HindIII-XhoI fragment of the human CaR cDNA was subcloned into the mammalian expression vector pcDNA3.1/Hygro (+) (Invitrogen) containing the hygromycin resistance gene as a selectable marker. This plasmid was transfected into HEK 293 cells by lipofection. The transfected cells were grown in DMEM containing 10% fetal bovine serum and hygromycin B (50 mg/mL) in 96 well plate. Hygromycin-resistant colonies were subcloned and assayed for hCaR mRNA by quantitative RT-PCR. The stably transfected cell line was then selected and used for agonistic activity studies.

Evocalcet was dissolved in dimethyl sulfoxide (DMSO) and diluted with HEPES buffer for the preparation of cells. Evocalcet solutions (3~30000 nM, 9-point concentrations) were added to a 96-well plate, and a suspension of HEK293 cells stably transfected with the human CaR was then seeded onto the plate. The ratio with the fluorescence intensity was determined continuously with a multi-label plate reader. To evaluate the agonistic activity pattern of evocalcet, calcium solutions in each concentration were added after the addition of evocalcet solutions to the plate.

### Pharmacodynamics and pharmacokinetics studies

All animal studies was carried out in strict accordance with the Standards for Proper Conduct of Animal Experiments at Kyowa Hakko Kirin Co., Ltd. The protocol was approved by the Institutional Animal Care and Use Committee (IACUC) of the Kyowa Hakko Kirin Co., Ltd. Male Sprague-Dawley rats (6–7 weeks of age) were purchased from CLEA Japan, Inc. (Shizuoka, Japan) or Charles River Laboratories Japan, Inc. (Kanagawa, Japan) for normal and 5/6 Nx rats studies. Male Wistar rats (5 weeks of age) were purchased from Charles River Laboratories Japan, Inc. (Kanagawa, Japan) for the evaluation of gastric emptying in rats. The rats were allowed free access to FR-2 diets (Funabashi Farm Co., Ltd., Chiba, Japan) and water. Female common marmosets (about 22–24 months of age) were purchased from CLEA Japan, Inc. (Gifu, Japan) for the evaluation of emesis. The marmosets had access to CMS-1M diet (CLEA Japan, Inc., Tokyo, Japan). All rats in the pharmacodynamics studies were euthanized by carbon dioxide inhalation after the completion of studies. Euthanasia by carbon dioxide inhalation was conducted in the home cage. An optimal flow rate is 20% replacement of the home cage volume/min. We observed the respiratory and cardiac arrest in rats, and maintained CO_2_ flow for at least 3 minutes after respiratory and cardiac arrest. After both signs were observed, rats were removed from the cage. In marmoset study, blood samples were collected from the femoral or saphenous vein without an anesthesia. IACUC approved the blood sampling of marmosets without the administration of anesthesia, analgesics, and antibiotics. Also, blood was collected aseptically. The blood sampling without an anesthesia was in accordance with Japanese marmoset textbook "Marmoset breeding / experiment technique / dissecting organization" (Adsley, Yoshikuni Tanioka, 1996). Furthermore, blood sampling was adequately performed using the restraint device for marmosets. The marmosets were acclimatized and trained to the restraint device. The cleaning of the restraint device was conducted every time. The marmoset was moved one by one from the bleeding room to other treatment room for the blood sampling to reduce social stress. Since the blood sample volume was small, it was considered that the restraint time was short and the stress caused by blood sampling was minimal. Based on the above reasons, the IACUC approval was obtained for blood sampling without anesthesia. The marmosets were excluded after the completion of study, and the euthanasia was not applied, and the marmosets were pooled.

#### Normal rat study

After acclimatization, rats were divided into nine groups (n = 10/group) matched in terms of body weight, and their serum PTH and calcium levels. Vehicle, 0.5% (w/v) methyl cellulose (Wako Pure Chemical Industries, Ltd.) solution orally administered to vehicle treatment group rats. Evocalcet (0.03, 0.1, 0.3, or 1 mg/kg), or cinacalcet (1, 3, 10, or 30 mg/kg) were suspended in the vehicle solution and orally administered to each drug treatment group. Blood samples were collected from the tail vein before and at 0.5, 2, 4, 6, 8, and 24 h after the oral administration.

#### 5/6 nephrectomized rats study

Rats were 5/6 nephrectomized in two steps. Under anesthesia (pentobarbital, 50 mg/kg; intraperitoneally) and analgesia (lidocaine; topically), two-thirds of the left kidney was removed, and then the right kidney was removed after a seven-day interval. Seven days after the completion of 5/6 nephrectomy, the FR-2 diet was changed to a high-phosphate diet, containing 0.6% calcium and 0.9% phosphate (Oriental Yeast Co., Ltd., Tokyo, Japan). Approximately 2 weeks after the initiation of the high-phosphate diet, the 5/6 Nx rats were divided into five groups (n = 12/group) matched in terms of their body weight as well as their blood urea nitrogen (BUN) and serum PTH and calcium levels.

In the single-dose study, blood samples were collected from the tail vein before and at 0.5, 2, 4, 8, 24, 48, and 72 h after the oral administration of vehicle (0.5% methyl cellulose solution) or evocalcet (0.03, 0.1, 0.3, or 1 mg/kg). In the repeated-dose study, vehicle or evocalcet was orally administered to the respective groups once daily for 14 days. Blood samples were obtained from the tail vein before the first administration and after the seventh and last administrations.

#### Biochemical analyses

The serum PTH level was measured using a Rat Intact PTH ELISA kit (Immutopics, Inc., San Clemente, CA). The serum calcium, phosphate and BUN levels were measured using an auto analyzer (Hitachi High-Technologies Corporation., Tokyo, Japan) or the following test kits: the Calcium E-test WAKO, the Phospha C-test WAKO, or the Blood Urea nitrogen B-test WAKO (Wako Pure Chemical Industries, Ltd., Osaka, Japan).

#### Pharmacokinetic analyses

The plasma concentrations of evocalcet were determined after single or repeated oral administration in both normal and 5/6 Nx rats. The pharmacokinetic (PK) parameters of the rat plasma concentrations were calculated individually using Phoenix WinNonlin 6.2, a PK analysis software program (Pharsight Corporation, Sunnyvale, CA).

#### Evaluation of gastric emptying in rats

The study of gastric emptying in rats was performed by referencing a previously reported method [25]. After acclimatization, rats were fasted for 20–26 h before administration and deprived of water from 4–10 h before administration. The rats were divided into eight body-weight-matched groups (n = 8/group). Vehicle (0.5% methyl cellulose solution), evocalcet (0.3, 1, or 3 mg/kg) or cinacalcet (10, 30, or 100 mg/kg) was orally administered to rats. Ten minutes later, 0.05 w/v% phenol red, a non-absorbable marker solution (1.5 mL/body), was orally administered. Thirty minutes after the administration of phenol red, the rats were euthanized by cervical dislocation, and the gastric body clamped at the cardiac and pyloric part of the stomach was obtained. The stomach was homogenized by adding 0.1 M NaOH and centrifugated. The supernatant was mixed with 20 w/v% trichloroacetic acid. After centrifugation, the supernatant was mixed with the same volume of 0.5 mol/L NaOH, and the absorbance was measured at 560 nm. The gastric emptying rate was calculated by the following formula:

$$\begin{array}{l} Gastric\;emptying\;rate\\ \;=[1\\ \;-(optical\;density\;[OD]\;value\;in\;each\;rat)\\ \;/\;(mean\;value\;of\;OD\;in\;the\;baseline\;group)]\\ \;\times 100 \end{array}$$

#### Evaluation of emesis in common marmosets

The study of effect of evocalcet on emesis in common marmoset was performed by referencing a previously reported method [26]. The marmosets (n = 24) used for this study were housed in cages (460 × 600 × 655 mm) with environmental enrichments (wooden perches, swing, and balls) and acclimated for at least 4 weeks. The marmosets were fasted from evening on the day before administration. To confirm the reduction in the serum PTH levels, blood samples (about 300~400 μL, 1.2∼1.6% of total blood volume) were collected from the femoral or saphenous vein without an anesthesia before and at 1 and 4 h after the oral administration of evocalcet (1.5 or 5 μg/kg, n = 3 in each dose) or cinacalcet (300 or 500 μg/kg, n = 3 in each dose) suspended in 0.5% methyl cellulose solution using a metallic gastric zonde. The serum PTH level was measured using a Porcine Intact PTH ELISA kit (Immutopics, Inc., San Clemente, CA, USA). To evaluate the emetic effects in marmosets, evocalcet (50 or 150 μg/kg) or cinacalcet (1500 or 5000 μg/kg) was orally administered (n = 6/drug, dose down titration, n = 12 in total). The observation of emesis was conducted until 4 h after the administration.

#### CYP inhibition assay

The direct inhibition of evocalcet (0.15~50 μM, 6-point concentrations) against the specific activities of 9 CYP isozymes (CYP1A2, CYP2A6, CYP2B6, CYP2C8, CYP2C9, CYP2C19, CYP2D6, CYP2E1, and CYP3A4/5) was examined in human liver microsomes without the pre-incubation of evocalcet, in the presence of the NADPH-generating system. Each CYP substrate was incubated with human liver microsomes in the absence or presence of evocalcet, and the inhibition of evocalcet for each CYP isozyme was measured.

#### Statistical analyses

The statistical analyses were all performed using the SAS software program (Release 9.2, SAS Institute Inc., Cary, NC). The differences in the mean values of two groups were determined by Fisher’s t test followed by Student’s t-test or the Aspin-Welch test. Since there were significant differences in the variance in multiple comparisons by the Bartlett test, the inter-group differences in probability distributions were determined using the Kruskal-Wallis test followed by the Steel test. Since there were no significant differences in the variance determined by the Bartlett test, the differences in the mean values were determined by a one-way ANOVA followed by the Dunnett test. P values of <0.05 were considered to indicate statistical significance in all of the analyses.

## Results

### Agonistic activity on the human calcium receptor

**Fig 2:**
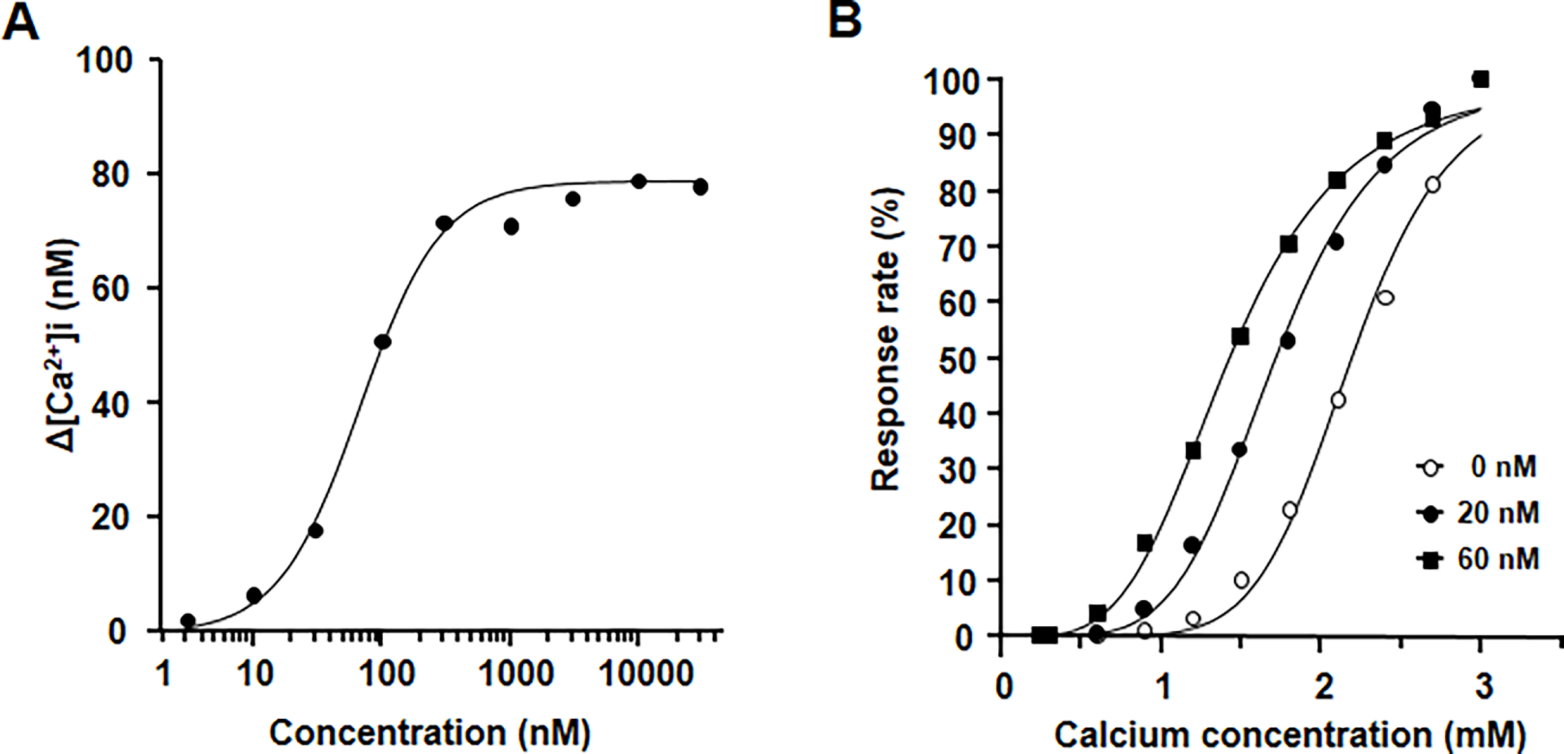
The agonistic activity of evocalcet on hCaR-expressing HEK293 (hCaR-HEK293) cells. The transient changes in the cytoplasmic calcium concentration (Δ[Ca^2+^]i) induced by receptor activation were measured. (A) The effect of evocalcet on the Δ[Ca^2+^]i value in hCaR-HEK293 cells. (B) The effect of evocalcet on the response rates elicited by increasing the extracellular calcium level in hCaR-HEK293 cells. The response rate was calculated using the following formula:
$$Response\;rate\;(\%)=\Delta [{Ca}^{2+}]i/Max\Delta [{Ca}^{2+}]i\times 100$$
Δ[Ca^2+^]i: Subtraction of basal [Ca^2+^]i from maximum [Ca^2+^]i observed after addition of evocalcet; Max Δ[Ca^2+^]i: Δ[Ca^2+^]i at the maximum Ca concentration (3 mmol/L).

The stimulation of CaR induces the intracellular release of calcium from cytoplasmic Ca stores. To confirm the agonistic action of evocalcet on human CaR (hCaR), the cytoplasmic Ca^2+^ concentrations ([Ca^2+^]i) were examined in HEK293 cells stably expressing hCaR (hCaR-HEK293). Evocalcet evoked concentration-dependent increases in [Ca^2+^]i (Fig 2A). The EC_50_ of evocalcet for [Ca^2+^]i was 92.7 nM. We also investigated the effects of evocalcet on the [Ca^2+^]i that were elicited by increasing the extracellular calcium concentration. When the concentration of evocalcet was increased, the concentration-response curves shifted to a lower range of extracellular calcium concentration (Fig 2B).

### Effects of evocalcet in normal rats

**Fig 3:**
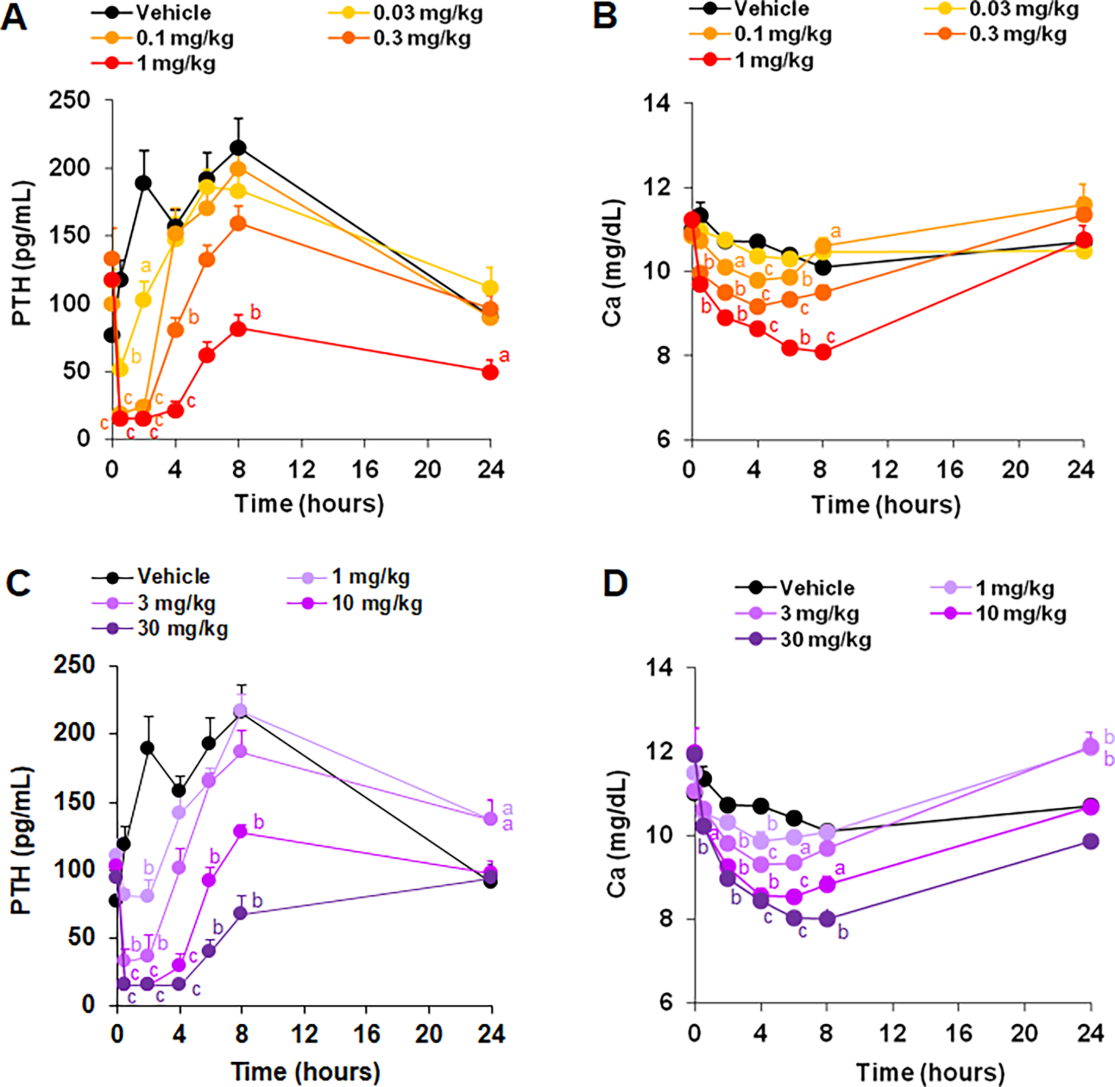
**The pharmacological effects of evocalcet and cinacalcet on the serum PTH (A; evocalcet, C; cinacalcet) and calcium (B; evocalcet, D; cinacalcet) levels in normal rats.** Vehicle, evocalcet (0.03, 0.1, 0.3, or 1 mg/kg), or cinacalcet (1, 3, 10, or 30 mg/kg) were orally administered to the rats. The data are presented as the mean + S.E. n = 10/group. ^a^*P* < 0.05, ^b^*P* < 0.01, and ^c^*P* < 0.001 vs. Vehicle group (Steel test).

To evaluate the effects on serum PTH and calcium levels, rats were orally treated with evocalcet or cinacalcet. Evocalcet significantly reduced the serum PTH level at doses of ≥0.03 mg/kg and the serum calcium level at doses of ≥0.1 mg/kg compared with the vehicle group (Fig 3A and 3B). Cinacalcet also significantly decreased the serum PTH and calcium levels at doses of ≥1 mg/kg compared with the vehicle group (Fig 3C and 3D). These results suggest that evocalcet exerted an approximately 30 times stronger pharmacological effect than cinacalcet at the same dosage in normal rats. The plasma concentrations of evocalcet in normal rats that received a single dose were measured to evaluate its pharmacokinetics (Table 1).

**Table 1:** Pharmacokinetic parameters of evocalcet after oral administration to male rats.

Dose	C_max_			*t*_max_			AUC_0-∞_			*t*_1/2_			*F*
(mg/kg)	(ng/mL)			(h)			(ng⋅h/mL)			(h)			(%)
0.1	222.2	±	19.8	0.25	±	0.00	819	±	262	6.66	±	1.94	81.6
0.3	599.6	±	59.8	0.38	±	0.14	2578	±	403	5.77	±	0.33	85.6
1	2188	±	291	0.81	±	0.38	8445	±	1108	6.13	±	0.61	84.2

The mean ± S.D. n = 4/group. F, bioavailability

### Effects of evocalcet in 5/6 Nx rats

**Fig 4:**
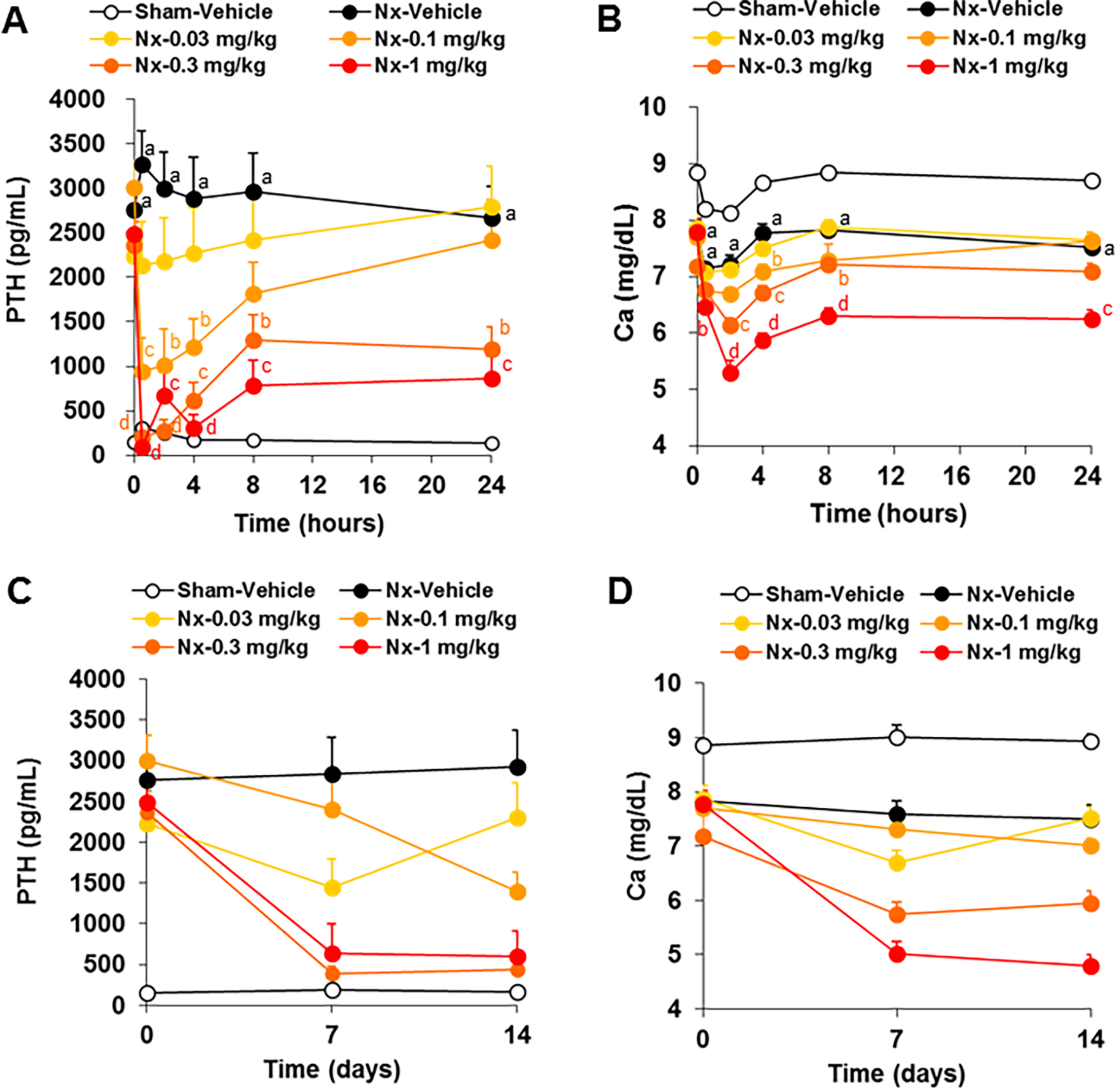
The pharmacological effects of a single and repeated dose of evocalcet in 5/6 Nx rats. Vehicle or evocalcet (0.03, 0.1, 0.3, or 1 mg/kg) was orally administered to sham-operated and 5/6 Nx rats. A and B are the time courses of serum PTH (A) and Ca (B) levels after the first administration. C and D are the serum PTH (C) and Ca (D) levels before (day 0) and 24 h after evocalcet administration (days 7 and 14). The data are presented as the mean + S.E. n = 12/group. ^a^*P* < 0.001 vs. Sham-vehicle group (Student’s *t*-test or Aspin-Welch test). ^b^*P* < 0.05, ^c^*P* < 0.01, and ^d^*P* < 0.001 vs. 5/6 Nx-vehicle group (Steel test).

To evaluate the effects of evocalcet on the serum PTH and calcium levels in 5/6 Nx rats, animals were treated with evocalcet. In the 5/6 Nx-vehicle group, the serum PTH levels were significantly increased, and the serum calcium levels were significantly decreased compared with the sham-vehicle group. Treatment with evocalcet significantly decreased both the serum PTH and calcium levels at doses of ≥0.1 mg/kg compared with the vehicle group (Fig 4A and 4B). To evaluate the long-term effects of evocalcet on the serum PTH and calcium levels in 5/6 Nx rats, animals were treated with evocalcet once daily for 14 days. Evocalcet (at ≥0.3 mg/kg) steadily decreased both the serum PTH and calcium levels at 24 h after administration on both days 7 and 14 (Fig 4C and 4D). The plasma concentrations of evocalcet were determined after repeated oral administration for 14 days in 5/6 Nx rats (Table 2). The mean plasma concentrations at 0.5 and 24 h increased in a dose-dependent manner and reached a stable trough level on days 7 and 14.

**Table 2:** Plasma concentrations of evocalcet after repeated oral administration to 5/6 nephrectomized rats.

	Plasma concentration (ng/mL)														
Dose	Day 1						Day 7			Day 14					
(mg/kg)	0.5 h			24 h			24 h			0.5 h			24 h		
0.03	64.96	±	14.58	4.696	±	2.861	6.838	±	6.788	51.46	±	24.48	9.081	±	6.781
0.1	204.2	±	64.1	21.78	±	16.45	53.46	±	41.95	133.8	±	71.2	29.24	±	29.53
0.3	545.5	±	154.9	90.48	±	65.16	190.6	±	105.3	686.9	±	310.2	102.1	±	97.1
1	1635	±	733	285.7	±	196.8	557.2	±	460.0	1769	±	1109	490.6	±	402.2

The mean ± S.D. n = 12/group.

### Effects of evocalcet on gastric emptying in rats

**Fig 5:**
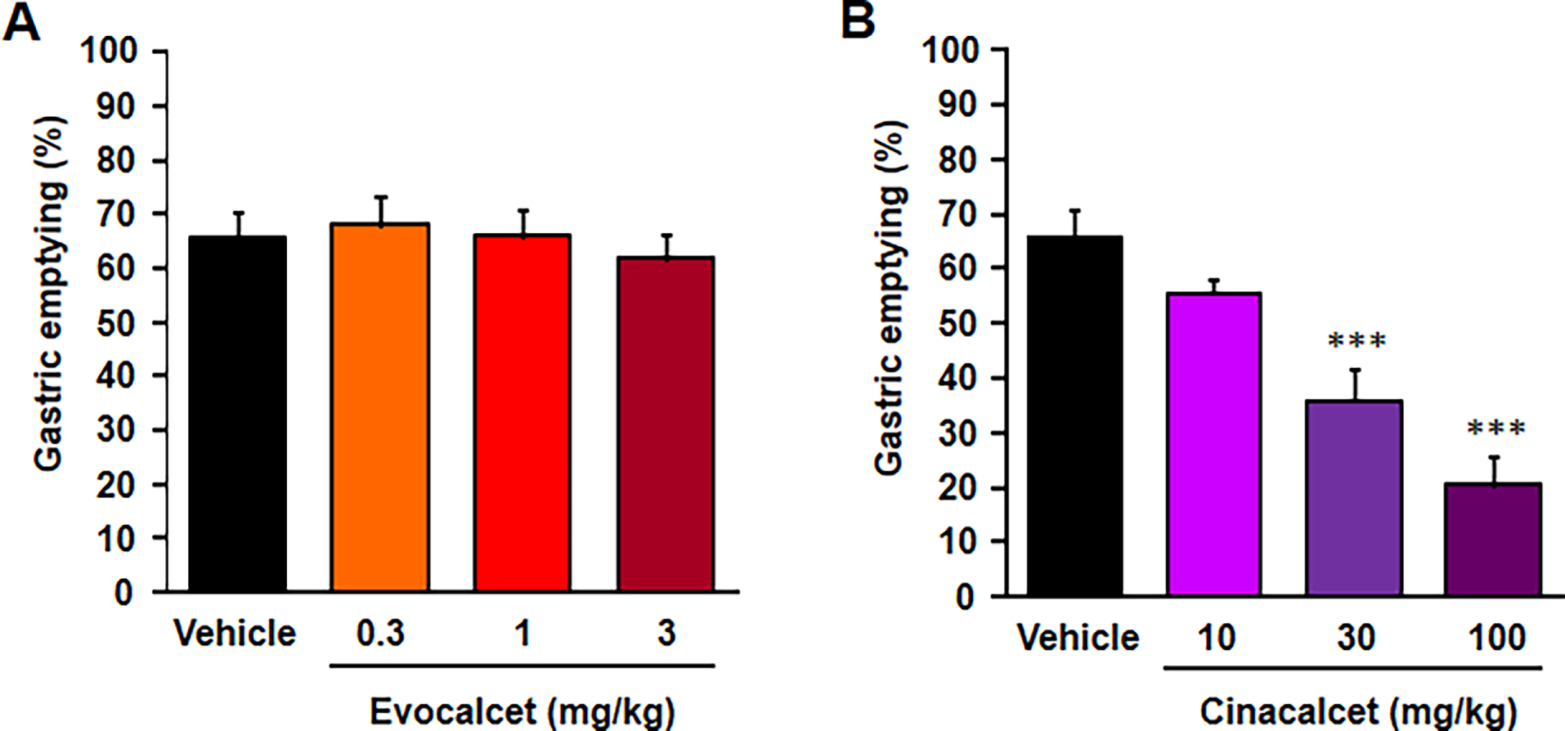
The effect of evocalcet on gastric emptying for 30 minutes in rats. Vehicle, evocalcet (0.3, 1, or 3 mg/kg), or cinacalcet (10, 30, or 100 mg/kg) were orally administered to rats. The data are presented as the mean + S.E. n = 8/group. ***P < 0.001 vs. vehicle group (Dunnett’s test).

To evaluate the direct effects of evocalcet on the GI tract, rats were treated with evocalcet (0.3, 1, and 3 mg/kg) or cinacalcet (10, 30, and 100 mg/kg), and the gastric emptying ratios were observed. Evocalcet had no significant effects on gastric emptying at any dose (Fig 5A). In contrast, cinacalcet caused a significant delay in gastric emptying dose-dependently at doses of ≥30 mg/kg (Fig 5B).

### Effects of evocalcet on emesis in common marmosets

To confirm the effective dose on serum PTH levels in marmosets, animals were treated with evocalcet (1.5 or 5 μg/kg) or cinacalcet (300 or 500 μg/kg). Evocalcet and cinacalcet effectively reduced the serum PTH levels in marmosets at 5 and 500 μg/kg, lower effective doses than those observed in rats, respectively. To assess the emetic effects, 6 marmosets were treated with evocalcet (50 and 150 μg/kg) or cinacalcet (1500 and 5000 μg/kg). Evocalcet caused vomiting in only 1 out of 6 marmosets at 150 μg/kg, while cinacalcet caused vomiting in 5 out of 6 marmosets at 5000 μg/kg, suggesting the less effects of evocalcet on emesis. In addition, evocalcet appears to have a wider safety window calculated by the doses achieving pharmacological efficacy (PTH suppression) and toxic effect (emesis) (Table 3).

**Table 3:** The effects of evocalcet and cinacalcet on emesis in common marmosets.

Treatment	Minimum effective dose on PTH reduction (μg/kg)	Dosage (μg/kg)	Number of emesis	Safety margin
Evocalcet	5	50	0/6	10 < X < 30
		150	1/6	
Cinacalcet	500	1500	0/6	3 < X < 10
		5000	5/6	

### CYP inhibition assay

The direct inhibitory effects of evocalcet on the activities of CYP isozymes were examined in human liver microsomes. Evocalcet showed no significant inhibitory effects on the specific activities of any CYP isozymes other than CYP2D6. The CYP2D6 activity decreased to 50.7% in the presence of 50 μM evocalcet; however, all of the IC_50_ values for the direct inhibition of the specific activities of the 9 CYP isozymes by evocalcet were higher than 50 μM.

## Discussion

SHPT is a common mineral metabolism abnormality in patients with CKD, especially those on maintenance dialysis. In sharp contrast to vitamin D receptor activators, it has been shown that cinacalcet effectively suppressed the PTH levels in patients with severe SHPT without increasing the serum Ca levels. Nevertheless, cinacalcet induced adverse events in the GI tract, which often result in poor adherence and insufficient dosing. Furthermore, drug-drug interactions also caused critical concerns because cinacalcet inhibits the CYP2D6 enzyme. Recently developed calcimimetic agent for intravenous use, etelcalcetide, may be a solution, especially for low adherance, however, in head to head study, it evoked GI events as frequent as by cincalcet [27]. Thus, there is an unmet need for a calcimimetic agent with less GI events and fewer drug-drug interaction [28]. Evocalcet is a newly synthesized calcimimetic compound for oral administration designed with the aim of alleviating these adverse effects of cinacalcet.

In our *in vitro* study, evocalcet evoked concentration-dependent increases in the cytoplasmic Ca^2+^ concentrations on hCaR-HEK293 cells. The concentration-response curves of evocalcet shift toward the lower extracellular calcium concentration range, and evocalcet causes no increase in the intracellular calcium concentration at the lowest calcium concentration, suggesting that this agent has the characteristics of an allosteric modulator of CaR.

Evocalcet suppressed the serum PTH and calcium levels in both normal and 5/6 Nx rats. This pharmacological profile is similar to that of cinacalcet. Of further note, evocalcet effectively reduced the serum PTH levels at a lower dosage than cinacalcet in rats. The bioavailability of evocalcet in rats was more than 80%, although that of cinacalcet in rats is approximately 1%-2%. It is suggested that such a higher bioavailability contributed to the reduction in the pharmacologically effective dose of evocalcet.

Evocalcet showed several advantages over cinacalcet concerning adverse effects. The main adverse effects associated with cinacalcet are GI events, such as nausea, vomiting, and abdominal discomforts [18]. It has also been reported that cinacalcet inhibited gastric emptying in hemodialysis patients who developed GI events following its treatment [21]. Cancer chemotherapeutic agents are known to cause GI symptoms and have been shown to induce delayed gastric emptying in humans, similar to cinacalcet treatment [29,30]. We therefore hypothesized that abnormal GI motility might be a good marker of the side effects in the GI tract. Cinacalcet inhibited gastric emptying in rats at a dose that was approximately 30-fold that required to achieve a significant PTH reduction. In contrast, evocalcet did not delay gastric emptying even at a dose of 3 mg/kg, which is 100-fold that required to achieve a significant PTH reduction. These data suggested that—in comparison to cinacalcet—evocalcet has less of an effect on GI tract motility.

We also used common marmosets to observe the emetic effects of both drugs. In comparison to cinacalcet, evocalcet induced emesis in fewer animals. These results suggested that evocalcet seemed to have a wider safety margin for emesis than cinacalcet.

The differences in the effects of evocalcet and cinacalcet on GI tracts may be due to the low exposure of the GI tract by a lower dosage of evocalcet than cinacalcet being used. Although the precise mechanism underlying the nausea and emesis observed with cinacalcet is still unknown, the less-marked effects of evocalcet on the GI tract seem to have contributed to the reduction in GI events compared to cinacalcet. Such a possibility remains to be elucidated in the near future.

Cinacalcet inhibits CYP2D6, and the co-administration of cinacalcet and dextromethorphan, an *in vivo* probe of the CYP2D6 activity, significantly increased the exposure of dextromethorphan in healthy human subjects [22,31]. In this study, we verified that the IC_50_ values of evocalcet against some CYP isozymes were >50 μM; evocalcet did not show substantial potency of tested CYP isozymes. Consequently, we showed that evocalcet is associated with a lower risk of a drug-drug interaction than cinacalcet.

In conclusion, the present study confirmed the following in animals: (1) based on its pharmacological profile, evocalcet is an allosteric modulator on parathyroid cells, which suppresses the serum levels of PTH; (2) evocalcet had a markedly milder effect on the GI tract than cinacalcet, strongly suggesting that evocalcet will have less GI adverse effects; and (3) evocalcet improved the pharmacokinetic profile. These findings suggest that evocalcet is an effective oral calcimimetic compound with a wide safety margin that can be used in the treatment of CKD with SHPT.

## Supporting information

S1 Table The set of raw data for Fig 2. (DOCX)

S2 Table The set of raw data for Fig 3. (DOCX)

S3 Table The set of raw data for Fig 4. (DOCX)

S4 Table The set of raw data for Fig 5. (DOCX)

S5 Table The set of raw data for Table 1. (DOCX)

S6 Table The set of raw data for Table 2. (DOCX)

S7 Table The set of raw data for Table 3. (DOCX)
