# Supplementary material for: A novel calcimimetic agent, evocalcet (MT-4580/KHK7580), suppresses the parathyroid cell function with little effect on the gastrointestinal tract or CYP isozymes in vivo and in vitro
Source: PLoS One. 2018 Apr 3;13(4):e0195316. doi: 10.1371/journal.pone.0195316 (PMC5882164; doi:10.1371/journal.pone.0195316)
Supplement: S1 Table — (DOCX) [file pone.0195316.s001.docx]

**S1 Table. The set of raw data for Fig. 2**

(A) The effect of evocalcet on the Δ[Ca^2+^]i value in hCaR-HEK293 cells.

| Concentration (nmol/L) | | | | | | | | |
| --- | --- | --- | --- | --- | --- | --- | --- | --- |
| 3 | 10 | 30 | 100 | 300 | 1000 | 3000 | 10000 | 30000 |
| Δ[Ca^2+^]i (nmol/L) | | | | | | | | |
| 1.94 | 6.18 | 17.71 | 50.62 | 71.35 | 70.70 | 75.59 | 78.61 | 77.66 |

(B) The effect of evocalcet on the response rates elicited by increasing the extracellular calcium level in hCaR-HEK293 cells.

| Evocalcet  (nmol/L) | Ca concentration (mmol/L) | | | | | | | | | | |
| --- | --- | --- | --- | --- | --- | --- | --- | --- | --- | --- | --- |
|  | 0.25 | 0.3 | 0.6 | 0.9 | 1.2 | 1.5 | 1.8 | 2.1 | 2.4 | 2.7 | 3.0 |
|  | Response rate (%) | | | | | | | | | | |
| 0 | 0 | 0 | 0 | 0.52 | 2.74 | 9.81 | 22.48 | 42.04 | 60.55 | 80.83 | 100 |
| 20 | 0 | 0 | 0.26 | 4.69 | 16.07 | 33.31 | 52.75 | 70.47 | 84.37 | 94.54 | 100 |
| 60 | 0 | 0 | 3.76 | 16.86 | 33.15 | 53.86 | 70.14 | 81.77 | 88.98 | 92.99 | 100 |
