# Supplementary material for: A novel calcimimetic agent, evocalcet (MT-4580/KHK7580), suppresses the parathyroid cell function with little effect on the gastrointestinal tract or CYP isozymes in vivo and in vitro
Source: PLoS One. 2018 Apr 3;13(4):e0195316. doi: 10.1371/journal.pone.0195316 (PMC5882164; doi:10.1371/journal.pone.0195316)
Supplement: S2 Table — (DOCX) [file pone.0195316.s002.docx]

**S2 Table. The set of raw data for Fig. 3**

(A) Serum PTH (evocalcet)

| **Group** | **Rat No.** | **Time (h)** | | | | | | |
| --- | --- | --- | --- | --- | --- | --- | --- | --- |
|  |  | **0** | **0.5** | **2** | **4** | **6** | **8** | **24** |
| **Vehicle** | **1-1** | 56.346 | 120.034 | 356.361 | 197.207 | 337.767 | 363.973 | 135.171 |
|  | **1-2** | 15.500 | 115.983 | 138.771 | 160.485 | 189.429 | 219.386 | 90.289 |
|  | **1-3** | 46.966 | 68.397 | 181.914 | 168.646 | 160.445 | 137.745 | 90.827 |
|  | **1-4** | 37.373 | 115.127 | 134.370 | 134.743 | 200.583 | 142.461 | 79.876 |
|  | **1-5** | 183.214 | 92.647 | 177.730 | 128.882 | 208.806 | 212.096 | 91.904 |
|  | **1-6** | 67.371 | 62.064 | 127.444 | 132.517 | 104.920 | 174.963 | 70.540 |
|  | **1-7** | 60.713 | 148.906 | 125.497 | 100.247 | 186.254 | 208.752 | 78.170 |
|  | **1-8** | 136.390 | 181.315 | 275.414 | 193.572 | 198.140 | 182.853 | 105.908 |
|  | **1-9** | 74.101 | 80.779 | 210.339 | 141.568 | 147.826 | 283.361 | 68.296 |
|  | **1-10** | 91.213 | 191.471 | 161.497 | 215.531 | 190.406 | 223.502 | 93.251 |
|  | **Mean** | **76.919** | **117.672** | **188.934** | **157.340** | **192.458** | **214.909** | **90.423** |
|  | **SE** | **15.655** | **14.140** | **23.616** | **11.485** | **18.919** | **21.310** | **6.145** |
| **0.03 mg/kg** | **2-1** | 129.159 | 33.535 | 15.500 | 111.671 | 167.854 | 144.862 | 85.800 |
|  | **2-2** | 98.086 | 36.787 | 69.801 | 66.789 | 143.511 | 141.432 | 128.079 |
|  | **2-3** | 98.874 | 59.553 | 132.927 | 176.435 | 260.179 | 139.974 | 93.520 |
|  | **2-4** | 128.658 | 50.196 | 117.200 | 179.922 | 212.225 | 226.246 | 115.512 |
|  | **2-5** | 81.261 | 64.403 | 119.941 | 200.545 | 211.330 | 213.297 | 45.316 |
|  | **2-6** | 126.366 | 70.451 | 109.480 | 117.161 | 175.263 | 177.022 | 85.890 |
|  | **2-7** | 104.386 | 74.902 | 83.508 | 137.784 | 176.565 | 177.107 | 75.657 |
|  | **2-8** | 122.715 | 34.333 | 88.630 | 93.718 | 131.380 | 148.207 | 215.959 |
|  | **2-9** | 112.906 | 15.500 | 158.250 | 165.530 | 164.597 | 214.155 | 131.939 |
|  | **2-10** | 170.470 | 80.322 | 138.699 | 226.956 | 221.669 | 253.260 | 141.454 |
|  | **Mean** | **117.288** | **51.998** | **103.394** | **147.651** | **186.457** | **183.556** | **111.913** |
|  | **SE** | **7.747** | **6.748** | **12.951** | **16.002** | **12.420** | **12.913** | **14.840** |

| **Group** | **Rat No.** | **Time (h)** | | | | | | |
| --- | --- | --- | --- | --- | --- | --- | --- | --- |
|  |  | **0** | **0.5** | **2** | **4** | **6** | **8** | **24** |
| **0.1 mg/kg** | **3-1** | 106.391 | 32.108 | 15.500 | 86.003 | 214.912 | 265.695 | 70.271 |
|  | **3-2** | 49.830 | 15.500 | 15.500 | 85.929 | 87.660 | 156.182 | 68.655 |
|  | **3-3** | 47.897 | 15.500 | 15.500 | 152.918 | 216.133 | 198.289 | 90.378 |
|  | **3-4** | 73.815 | 15.500 | 15.500 | 206.184 | 165.086 | 133.542 | 71.528 |
|  | **3-5** | 69.877 | 15.500 | 37.768 | 145.426 | 201.071 | 209.695 | 57.973 |
|  | **3-6** | 187.653 | 15.500 | 15.500 | 142.532 | 210.434 | 195.545 | 99.983 |
|  | **3-7** | 112.047 | 33.820 | 42.097 | 266.348 | 153.036 | 303.000 | 120.270 |
|  | **3-8** | 96.726 | 15.500 | 52.053 | 163.156 | 149.943 | 168.617 | 80.325 |
|  | **3-9** | 90.998 | 15.500 | 15.500 | 79.252 | 133.171 | 221.701 | 122.155 |
|  | **3-10** | 167.821 | 15.500 | 15.500 | 192.014 | 173.553 | 146.406 | 114.615 |
|  | **Mean** | **100.306** | **18.993** | **24.042** | **151.976** | **170.500** | **199.867** | **89.615** |
|  | **SE** | **14.676** | **2.332** | **4.484** | **18.822** | **13.168** | **16.900** | **7.424** |
| **0.3 mg/kg** | **4-1** | 152.571 | 15.500 | 15.500 | 82.739 | 164.109 | 120.507 | 37.956 |
|  | **4-2** | 98.372 | 15.500 | 15.500 | 66.121 | 114.364 | 151.294 | 121.437 |
|  | **4-3** | 164.312 | 15.500 | 15.500 | 90.380 | 121.692 | 188.770 | 108.511 |
|  | **4-4** | 282.517 | 15.500 | 15.500 | 92.754 | 189.592 | 230.534 | 117.308 |
|  | **4-5** | 95.007 | 15.500 | 15.500 | 118.645 | 139.033 | 160.899 | 43.342 |
|  | **4-6** | 72.383 | 15.500 | 15.500 | 49.726 | 100.931 | 177.793 | 118.295 |
|  | **4-7** | 220.945 | 15.500 | 15.500 | 54.474 | 153.606 | 194.259 | 85.531 |
|  | **4-8** | 91.786 | 15.500 | 15.500 | 71.463 | 78.216 | 97.095 | 122.873 |
|  | **4-9** | 87.776 | 15.500 | 15.500 | 130.440 | 132.031 | 142.547 | 115.153 |
|  | **4-10** | 66.154 | 15.500 | 15.500 | 54.400 | 132.846 | 133.371 | 90.558 |
|  | **Mean** | **133.182** | **15.500** | **15.500** | **81.114** | **132.642** | **159.707** | **96.096** |
|  | **SE** | **22.603** | **0.000** | **0.000** | **8.687** | **10.075** | **12.427** | **10.069** |

| **Group** | **Rat No.** | **Time (h)** | | | | | | |
| --- | --- | --- | --- | --- | --- | --- | --- | --- |
|  |  | **0** | **0.5** | **2** | **4** | **6** | **8** | **24** |
| **1 mg/kg** | **5-1** | 121.999 | 15.500 | 15.500 | 15.500 | 33.194 | 116.477 | 15.500 |
|  | **5-2** | 99.303 | 15.500 | 15.500 | 76.656 | 117.702 | 34.835 | 67.399 |
|  | **5-3** | 46.609 | 15.500 | 15.500 | 15.500 | 55.257 | 83.288 | 15.500 |
|  | **5-4** | 126.510 | 15.500 | 15.500 | 15.500 | 50.454 | 35.521 | 62.821 |
|  | **5-5** | 76.750 | 15.500 | 15.500 | 15.500 | 46.627 | 87.319 | 70.361 |
|  | **5-6** | 221.732 | 15.500 | 15.500 | 15.500 | 45.650 | 70.511 | 40.110 |
|  | **5-7** | 178.130 | 15.500 | 15.500 | 15.500 | 31.402 | 83.889 | 49.356 |
|  | **5-8** | 103.742 | 15.500 | 15.500 | 15.500 | 105.083 | 72.054 | 40.918 |
|  | **5-9** | 137.750 | 15.500 | 15.500 | 15.500 | 47.848 | 139.974 | 35.442 |
|  | **5-10** | 67.658 | 15.500 | 15.500 | 15.500 | 90.510 | 95.209 | 101.419 |
|  | **Mean** | **118.018** | **15.500** | **15.500** | **21.616** | **62.373** | **81.908** | **49.883** |
|  | **SE** | **16.580** | **0.000** | **0.000** | **6.116** | **9.672** | **10.205** | **8.366** |

(B) Serum Ca (evocalcet)

| **Group** | **Rat No.** | **Time (h)** | | | | | | |
| --- | --- | --- | --- | --- | --- | --- | --- | --- |
|  |  | **0** | **0.5** | **2** | **4** | **6** | **8** | **24** |
| **Vehicle** | **1-1** | 10.8 | 13.6 | 10.4 | 11.2 | 10.2 | 10.2 | 10.2 |
|  | **1-2** | 11.0 | 10.4 | 10.6 | 10.8 | 10.4 | 10.2 | 10.8 |
|  | **1-3** | 11.8 | 12.2 | 11.4 | 10.6 | 10.6 | 9.6 | 11.0 |
|  | **1-4** | 11.0 | 11.0 | 11.2 | 10.4 | 10.0 | 10.2 | 10.6 |
|  | **1-5** | 11.2 | 11.4 | 10.8 | 10.8 | 10.4 | 10.2 | 11.0 |
|  | **1-6** | 10.8 | 11.6 | 10.8 | 10.8 | 10.6 | 10.4 | 10.6 |
|  | **1-7** | 10.6 | 11.2 | 10.6 | 10.6 | 10.4 | 10.2 | 10.4 |
|  | **1-8** | 11.0 | 10.4 | 10.8 | 10.6 | 10.4 | 10.4 | 10.8 |
|  | **1-9** | 11.0 | 10.8 | 10.8 | 10.6 | 10.6 | 9.2 | 10.6 |
|  | **1-10** | 10.8 | 10.8 | 9.8 | 10.6 | 10.4 | 10.4 | 11.0 |
|  | **Mean** | **11.0** | **11.3** | **10.7** | **10.7** | **10.4** | **10.1** | **10.7** |
|  | **SE** | **0.1** | **0.3** | **0.1** | **0.1** | **0.1** | **0.1** | **0.1** |
| **0.03 mg/kg** | **2-1** | 10.2 | 10.2 | 10.0 | 10.2 | 10.0 | 9.6 | 10.2 |
|  | **2-2** | 11.0 | 10.6 | 11.2 | 10.8 | 10.4 | 10.0 | 10.4 |
|  | **2-3** | 11.2 | 11.8 | 10.6 | 10.0 | 10.0 | 8.6 | 10.2 |
|  | **2-4** | 10.8 | 10.4 | 10.8 | 10.2 | 10.2 | 10.6 | 10.6 |
|  | **2-5** | 11.0 | 10.6 | 11.0 | 10.6 | 10.4 | 11.4 | 10.8 |
|  | **2-6** | 11.0 | 11.2 | 11.2 | 10.2 | 10.2 | 10.6 | 10.8 |
|  | **2-7** | 10.8 | 11.2 | 10.4 | 10.4 | 10.4 | 9.6 | 10.8 |
|  | **2-8** | 11.0 | 10.4 | 11.0 | 10.4 | 10.6 | 10.8 | 10.2 |
|  | **2-9** | 10.8 | 11.8 | 10.8 | 10.4 | 10.2 | 10.8 | 10.2 |
|  | **2-10** | 11.2 | 11.4 | 10.6 | 10.6 | 10.6 | 12.6 | 10.6 |
|  | **Mean** | **10.9** | **11.0** | **10.8** | **10.4** | **10.3** | **10.5** | **10.5** |
|  | **SE** | **0.1** | **0.2** | **0.1** | **0.1** | **0.1** | **0.3** | **0.1** |

| **Group** | **Rat No.** | **Time (h)** | | | | | | |
| --- | --- | --- | --- | --- | --- | --- | --- | --- |
|  |  | **0** | **0.5** | **2** | **4** | **6** | **8** | **24** |
| **0.1 mg/kg** | **3-1** | 10.6 | 14.0 | 10.2 | 10.0 | 9.6 | 10.2 | 10.6 |
|  | **3-2** | 11.0 | 11.2 | 10.4 | 9.8 | 9.8 | 11.4 | 10.4 |
|  | **3-3** | 10.6 | 10.6 | 10.0 | 9.6 | 9.8 | 10.2 | 10.4 |
|  | **3-4** | 10.8 | 10.4 | 10.0 | 9.4 | 9.6 | 10.4 | 10.8 |
|  | **3-5** | 11.0 | 10.0 | 10.4 | 10.0 | 10.0 | 10.8 | 10.8 |
|  | **3-6** | 10.8 | 9.8 | 10.0 | 9.6 | 9.6 | 10.6 | 15.0 |
|  | **3-7** | 11.0 | 10.6 | 10.2 | 9.8 | 10.0 | 10.4 | 12.6 |
|  | **3-8** | 11.0 | 10.6 | 10.0 | 10.0 | 10.0 | 10.8 | 12.6 |
|  | **3-9** | 10.8 | 10.0 | 9.8 | 10.2 | 10.4 | 10.8 | 10.8 |
|  | **3-10** | 11.0 | 10.0 | 10.0 | 9.6 | 9.8 | 10.6 | 12.0 |
|  | **Mean** | **10.9** | **10.7** | **10.1** | **9.8** | **9.9** | **10.6** | **11.6** |
|  | **SE** | **0.1** | **0.4** | **0.1** | **0.1** | **0.1** | **0.1** | **0.5** |
| **0.3 mg/kg** | **4-1** | 10.4 | 9.6 | 9.0 | 8.8 | 8.6 | 9.8 | 14.8 |
|  | **4-2** | 10.8 | 9.4 | 9.2 | 9.6 | 9.8 | 10.4 | 11.4 |
|  | **4-3** | 11.2 | 10.4 | 9.4 | 9.4 | 9.8 | 9.8 | 11.0 |
|  | **4-4** | 10.8 | 9.6 | 9.8 | 9.8 | 9.6 | 9.8 | 11.0 |
|  | **4-5** | 11.2 | 10.4 | 9.8 | 9.2 | 9.4 | 9.4 | 11.2 |
|  | **4-6** | 10.8 | 10.0 | 9.4 | 9.0 | 9.0 | 8.8 | 10.4 |
|  | **4-7** | 10.6 | 10.0 | 9.4 | 9.0 | 9.2 | 8.4 | 10.8 |
|  | **4-8** | 11.2 | 10.4 | 9.8 | 9.2 | 9.6 | 9.8 | 11.0 |
|  | **4-9** | 11.2 | 9.8 | 9.6 | 8.8 | 9.2 | 9.6 | 11.4 |
|  | **4-10** | 11.0 | 10.0 | 9.6 | 9.0 | 9.2 | 9.2 | 10.6 |
|  | **Mean** | **10.9** | **10.0** | **9.5** | **9.2** | **9.3** | **9.5** | **11.4** |
|  | **SE** | **0.1** | **0.1** | **0.1** | **0.1** | **0.1** | **0.2** | **0.4** |

| **Group** | **Rat No.** | **Time (h)** | | | | | | |
| --- | --- | --- | --- | --- | --- | --- | --- | --- |
|  |  | **0** | **0.5** | **2** | **4** | **6** | **8** | **24** |
| **1 mg/kg** | **5-1** | 10.8 | 9.8 | 9.0 | 8.8 | 8.6 | 8.0 | 13.4 |
|  | **5-2** | 10.8 | 9.4 | 9.0 | 8.4 | 8.2 | 8.0 | 10.6 |
|  | **5-3** | 12.2 | 10.0 | 9.2 | 9.2 | 8.4 | 8.6 | 10.6 |
|  | **5-4** | 11.0 | 10.0 | 9.2 | 8.8 | 8.4 | 8.4 | 10.4 |
|  | **5-5** | 12.2 | 9.8 | 9.0 | 8.2 | 8.2 | 8.0 | 10.4 |
|  | **5-6** | 11.8 | 9.8 | 8.6 | 8.4 | 7.6 | 7.6 | 9.8 |
|  | **5-7** | 10.4 | 9.4 | 8.8 | 8.4 | 8.0 | 7.8 | 9.6 |
|  | **5-8** | 10.6 | 9.4 | 8.6 | 8.4 | 7.8 | 8.0 | 10.8 |
|  | **5-9** | 11.0 | 9.6 | 9.0 | 8.8 | 8.0 | 8.0 | 11.6 |
|  | **5-10** | 11.6 | 9.8 | 8.6 | 9.0 | 8.6 | 8.6 | 10.4 |
|  | **Mean** | **11.2** | **9.7** | **8.9** | **8.6** | **8.2** | **8.1** | **10.8** |
|  | **SE** | **0.2** | **0.1** | **0.1** | **0.1** | **0.1** | **0.1** | **0.3** |

(C) Serum PTH (cinacalcet)

| **Group** | **Rat No.** | **Time (h)** | | | | | | |
| --- | --- | --- | --- | --- | --- | --- | --- | --- |
|  |  | **0** | **0.5** | **2** | **4** | **6** | **8** | **24** |
| **Vehicle** | **1-1** | 56.346 | 120.034 | 356.361 | 197.207 | 337.767 | 363.973 | 135.171 |
|  | **1-2** | 15.500 | 115.983 | 138.771 | 160.485 | 189.429 | 219.386 | 90.289 |
|  | **1-3** | 46.966 | 68.397 | 181.914 | 168.646 | 160.445 | 137.745 | 90.827 |
|  | **1-4** | 37.373 | 115.127 | 134.370 | 134.743 | 200.583 | 142.461 | 79.876 |
|  | **1-5** | 183.214 | 92.647 | 177.730 | 128.882 | 208.806 | 212.096 | 91.904 |
|  | **1-6** | 67.371 | 62.064 | 127.444 | 132.517 | 104.920 | 174.963 | 70.540 |
|  | **1-7** | 60.713 | 148.906 | 125.497 | 100.247 | 186.254 | 208.752 | 78.170 |
|  | **1-8** | 136.390 | 181.315 | 275.414 | 193.572 | 198.140 | 182.853 | 105.908 |
|  | **1-9** | 74.101 | 80.779 | 210.339 | 141.568 | 147.826 | 283.361 | 68.296 |
|  | **1-10** | 91.213 | 191.471 | 161.497 | 215.531 | 190.406 | 223.502 | 93.251 |
|  | **Mean** | **76.919** | **117.672** | **188.934** | **157.340** | **192.458** | **214.909** | **90.423** |
|  | **SE** | **15.655** | **14.140** | **23.616** | **11.485** | **18.919** | **21.310** | **6.145** |
| **1 mg/kg** | **6-1** | 125.722 | 82.661 | 48.013 | 61.596 | 193.418 | 174.106 | 113.627 |
|  | **6-2** | 130.447 | 77.184 | 15.500 | 118.199 | 170.704 | 258.920 | 116.500 |
|  | **6-3** | 67.586 | 76.842 | 63.091 | 215.679 | 168.342 | 229.591 | 103.304 |
|  | **6-4** | 199.824 | 101.034 | 101.472 | 200.323 | 210.841 | 209.695 | 199.622 |
|  | **6-5** | 100.664 | 110.106 | 98.442 | 159.521 | 168.342 | 192.029 | 108.780 |
|  | **6-6** | 97.298 | 55.958 | 56.814 | 92.828 | 173.065 | 200.519 | 152.854 |
|  | **6-7** | 150.924 | 55.844 | 145.553 | 169.313 | 135.125 | 209.009 | 112.730 |
|  | **6-8** | 89.996 | 88.538 | 109.841 | 166.494 | 169.319 | 246.914 | 224.397 |
|  | **6-9** | 55.272 | 77.412 | 112.583 | 115.974 | 98.407 | 151.294 | 139.120 |
|  | **6-10** | 79.471 | 82.661 | 44.766 | 109.149 | 166.633 | 292.451 | 99.534 |
|  | **Mean** | **109.720** | **80.824** | **79.608** | **140.908** | **165.420** | **216.453** | **137.047** |
|  | **SE** | **13.655** | **5.391** | **12.604** | **15.519** | **9.647** | **13.195** | **13.625** |

| **Group** | **Rat No.** | **Time (h)** | | | | | | |
| --- | --- | --- | --- | --- | --- | --- | --- | --- |
|  |  | **0** | **0.5** | **2** | **4** | **6** | **8** | **24** |
| **3 mg/kg** | **7-1** | 128.085 | 15.500 | 15.500 | 103.659 | 183.160 | 189.714 | 196.839 |
|  | **7-2** | 105.317 | 95.614 | 15.500 | 15.500 | 174.856 | 304.715 | 94.418 |
|  | **7-3** | 83.480 | 15.500 | 15.500 | 90.677 | 149.373 | 168.703 | 114.256 |
|  | **7-4** | 135.602 | 15.500 | 15.500 | 42.604 | 151.001 | 164.415 | 163.088 |
|  | **7-5** | 85.557 | 49.739 | 178.523 | 174.729 | 143.429 | 139.889 | 110.127 |
|  | **7-6** | 97.370 | 38.955 | 15.500 | 129.105 | 187.556 | 219.300 | 122.065 |
|  | **7-7** | 56.632 | 15.500 | 42.818 | 141.716 | 155.316 | 158.155 | 109.678 |
|  | **7-8** | 140.686 | 15.500 | 15.500 | 112.265 | 207.503 | 206.865 | 94.507 |
|  | **7-9** | 84.483 | 54.018 | 32.934 | 135.633 | 155.316 | 189.885 | 141.634 |
|  | **7-10** | 109.613 | 15.500 | 15.500 | 62.560 | 133.171 | 126.682 | 226.462 |
|  | **Mean** | **102.683** | **33.133** | **36.278** | **100.845** | **164.068** | **186.832** | **137.307** |
|  | **SE** | **8.413** | **8.508** | **16.093** | **15.464** | **7.333** | **15.932** | **14.187** |
| **10 mg/kg** | **8-1** | 118.563 | 15.500 | 15.500 | 15.500 | 151.652 | 105.500 | 74.400 |
|  | **8-2** | 87.060 | 15.500 | 15.500 | 15.500 | 72.680 | 126.082 | 84.903 |
|  | **8-3** | 104.458 | 15.500 | 15.500 | 15.500 | 65.190 | 144.262 | 157.971 |
|  | **8-4** | 110.687 | 15.500 | 15.500 | 15.500 | 58.269 | 150.608 | 59.589 |
|  | **8-5** | 72.813 | 15.500 | 15.500 | 15.500 | 98.488 | 124.109 | 108.062 |
|  | **8-6** | 79.042 | 15.500 | 15.500 | 15.500 | 114.690 | 117.763 | 107.972 |
|  | **8-7** | 81.762 | 15.500 | 15.500 | 68.050 | 47.930 | 133.114 | 91.904 |
|  | **8-8** | 77.180 | 15.500 | 15.500 | 15.500 | 85.788 | 109.102 | 79.068 |
|  | **8-9** | 129.159 | 15.500 | 15.500 | 98.911 | 120.308 | 148.893 | 123.950 |
|  | **8-10** | 156.437 | 15.500 | 15.500 | 15.500 | 100.931 | 117.935 | 87.416 |
|  | **Mean** | **101.716** | **15.500** | **15.500** | **29.096** | **91.593** | **127.737** | **97.524** |
|  | **SE** | **8.618** | **0.000** | **0.000** | **9.351** | **10.113** | **5.084** | **8.932** |

| **Group** | **Rat No.** | **Time (h)** | | | | | | |
| --- | --- | --- | --- | --- | --- | --- | --- | --- |
|  |  | **0** | **0.5** | **2** | **4** | **6** | **8** | **24** |
| **30 mg/kg** | **9-1** | 138.251 | 15.500 | 15.500 | 15.500 | 66.737 | 15.500 | 171.526 |
|  | **9-2** | 87.633 | 15.500 | 15.500 | 15.500 | 15.500 | 114.590 | 66.411 |
|  | **9-3** | 57.420 | 15.500 | 15.500 | 15.500 | 15.500 | 49.500 | 119.552 |
|  | **9-4** | 75.748 | 15.500 | 15.500 | 15.500 | 15.500 | 15.500 | 71.977 |
|  | **9-5** | 90.783 | 15.500 | 15.500 | 15.500 | 103.210 | 109.616 | 90.917 |
|  | **9-6** | 139.898 | 15.500 | 15.500 | 15.500 | 15.500 | 121.536 | 84.274 |
|  | **9-7** | 94.578 | 15.500 | 15.500 | 15.500 | 37.101 | 84.403 | 76.016 |
|  | **9-8** | 106.964 | 15.500 | 15.500 | 15.500 | 65.027 | 89.892 | 88.314 |
|  | **9-9** | 69.848 | 15.500 | 15.500 | 15.500 | 44.384 | 59.843 | 92.298 |
|  | **9-10** | 80.387 | 15.500 | 15.500 | 15.500 | 15.500 | 15.500 | 80.562 |
|  | **Mean** | **94.151** | **15.500** | **15.500** | **15.500** | **39.396** | **67.588** | **94.185** |
|  | **SE** | **8.643** | **0.000** | **0.000** | **0.000** | **9.639** | **13.413** | **9.748** |

(D) Serum Ca (cinacalcet)

| **Group** | **Rat No.** | **Time (h)** | | | | | | |
| --- | --- | --- | --- | --- | --- | --- | --- | --- |
|  |  | **0** | **0.5** | **2** | **4** | **6** | **8** | **24** |
| **Vehicle** | **1-1** | 10.8 | 13.6 | 10.4 | 11.2 | 10.2 | 10.2 | 10.2 |
|  | **1-2** | 11.0 | 10.4 | 10.6 | 10.8 | 10.4 | 10.2 | 10.8 |
|  | **1-3** | 11.8 | 12.2 | 11.4 | 10.6 | 10.6 | 9.6 | 11.0 |
|  | **1-4** | 11.0 | 11.0 | 11.2 | 10.4 | 10.0 | 10.2 | 10.6 |
|  | **1-5** | 11.2 | 11.4 | 10.8 | 10.8 | 10.4 | 10.2 | 11.0 |
|  | **1-6** | 10.8 | 11.6 | 10.8 | 10.8 | 10.6 | 10.4 | 10.6 |
|  | **1-7** | 10.6 | 11.2 | 10.6 | 10.6 | 10.4 | 10.2 | 10.4 |
|  | **1-8** | 11.0 | 10.4 | 10.8 | 10.6 | 10.4 | 10.4 | 10.8 |
|  | **1-9** | 11.0 | 10.8 | 10.8 | 10.6 | 10.6 | 9.2 | 10.6 |
|  | **1-10** | 10.8 | 10.8 | 9.8 | 10.6 | 10.4 | 10.4 | 11.0 |
|  | **Mean** | **11.0** | **11.3** | **10.7** | **10.7** | **10.4** | **10.1** | **10.7** |
|  | **SE** | **0.1** | **0.3** | **0.1** | **0.1** | **0.1** | **0.1** | **0.1** |
| **1 mg/kg** | **6-1** | 14.6 | 10.4 | 10.4 | 8.0 | 9.6 | 10.0 | 12.2 |
|  | **6-2** | 10.4 | 10.4 | 10.4 | 10.2 | 9.4 | 10.4 | 11.8 |
|  | **6-3** | 11.2 | 10.4 | 10.2 | 9.8 | 10.0 | 10.0 | 11.2 |
|  | **6-4** | 11.2 | 10.8 | 10.4 | 9.8 | 10.0 | 10.2 | 12.6 |
|  | **6-5** | 10.8 | 10.0 | 10.0 | 10.4 | 9.6 | 9.8 | 11.4 |
|  | **6-6** | 12.0 | 10.8 | 10.6 | 10.4 | 10.4 | 10.4 | 13.0 |
|  | **6-7** | 11.0 | 10.6 | 10.2 | 10.0 | 10.2 | 10.0 | 12.6 |
|  | **6-8** | 11.2 | 10.2 | 10.0 | 9.8 | 10.0 | 10.0 | 11.0 |
|  | **6-9** | 11.4 | 10.8 | 10.2 | 10.0 | 10.2 | 10.0 | 12.6 |
|  | **6-10** | 11.0 | 10.8 | 10.6 | 10.2 | 10.2 | 10.0 | 12.4 |
|  | **Mean** | **11.5** | **10.5** | **10.3** | **9.9** | **10.0** | **10.1** | **12.1** |
|  | **SE** | **0.4** | **0.1** | **0.1** | **0.2** | **0.1** | **0.1** | **0.2** |

| **Group** | **Rat No.** | **Time (h)** | | | | | | |
| --- | --- | --- | --- | --- | --- | --- | --- | --- |
|  |  | **0** | **0.5** | **2** | **4** | **6** | **8** | **24** |
| **3 mg/kg** | **7-1** | 11.2 | 10.8 | 9.8 | 9.2 | 9.2 | 9.8 | 12.2 |
|  | **7-2** | 12.2 | 10.2 | 10.0 | 10.0 | 9.6 | 10.0 | 12.0 |
|  | **7-3** | 11.4 | 10.4 | 9.6 | 9.2 | 9.2 | 9.8 | 12.2 |
|  | **7-4** | 10.8 | 10.6 | 9.6 | 9.4 | 9.4 | 9.6 | 11.8 |
|  | **7-5** | 11.0 | 10.4 | 10.4 | 9.4 | 9.4 | 9.4 | 12.0 |
|  | **7-6** | 10.8 | 11.0 | 10.4 | 9.0 | 9.4 | 9.8 | 15.0 |
|  | **7-7** | 11.2 | 10.8 | 9.8 | 9.2 | 9.4 | 9.6 | 12.0 |
|  | **7-8** | 9.4 | 10.6 | 8.6 | 8.8 | 8.8 | 9.2 | 11.2 |
|  | **7-9** | 11.4 | 11.2 | 9.8 | 9.4 | 9.4 | 9.8 | 11.6 |
|  | **7-10** | 11.2 | 10.2 | 10.0 | 9.4 | 9.6 | 9.8 | 11.2 |
|  | **Mean** | **11.1** | **10.6** | **9.8** | **9.3** | **9.3** | **9.7** | **12.1** |
|  | **SE** | **0.2** | **0.1** | **0.2** | **0.1** | **0.1** | **0.1** | **0.3** |
| **10 mg/kg** | **8-1** | 9.6 | 10.0 | 8.6 | 8.2 | 8.0 | 8.6 | 11.4 |
|  | **8-2** | 11.4 | 10.2 | 9.6 | 9.2 | 8.8 | 10.4 | 11.6 |
|  | **8-3** | 11.4 | 10.8 | 9.4 | 8.8 | 8.6 | 8.4 | 11.0 |
|  | **8-4** | 10.6 | 9.8 | 9.2 | 8.2 | 8.6 | 8.8 | 10.2 |
|  | **8-5** | 11.2 | 10.2 | 9.0 | 8.6 | 8.6 | 8.6 | 10.4 |
|  | **8-6** | 12.0 | 10.4 | 9.4 | 9.2 | 8.6 | 9.0 | 10.4 |
|  | **8-7** | 15.0 | 10.4 | 9.0 | 8.6 | 8.8 | 8.6 | 10.4 |
|  | **8-8** | 11.8 | 11.0 | 9.8 | 7.4 | 8.2 | 8.4 | 10.4 |
|  | **8-9** | 15.6 | 10.2 | 9.2 | 8.4 | 8.4 | 8.6 | 10.6 |
|  | **8-10** | 11.0 | 9.2 | 9.2 | 9.0 | 8.8 | 8.8 | 10.4 |
|  | **Mean** | **12.0** | **10.2** | **9.2** | **8.6** | **8.5** | **8.8** | **10.7** |
|  | **SE** | **0.6** | **0.2** | **0.1** | **0.2** | **0.1** | **0.2** | **0.2** |

| **Group** | **Rat No.** | **Time (h)** | | | | | | |
| --- | --- | --- | --- | --- | --- | --- | --- | --- |
|  |  | **0** | **0.5** | **2** | **4** | **6** | **8** | **24** |
| **30 mg/kg** | **9-1** | 14.4 | 9.6 | 8.8 | 8.2 | 7.6 | 7.8 | 9.8 |
|  | **9-2** | 13.0 | 10.2 | 9.8 | 9.2 | 8.2 | 10.0 | 10.4 |
|  | **9-3** | 11.2 | 10.4 | 9.2 | 8.4 | 7.8 | 7.4 | 10.2 |
|  | **9-4** | 12.0 | 10.6 | 9.2 | 8.4 | 8.4 | 8.0 | 10.2 |
|  | **9-5** | 11.2 | 10.2 | 8.8 | 8.2 | 7.6 | 7.4 | 10.6 |
|  | **9-6** | 12.6 | 10.4 | 8.8 | 8.2 | 8.2 | 7.4 | 10.4 |
|  | **9-7** | 11.0 | 10.4 | 8.8 | 8.6 | 8.2 | 8.0 | 9.4 |
|  | **9-8** | 11.6 | 10.2 | 9.2 | 8.4 | 8.0 | 8.2 | 9.0 |
|  | **9-9** | 10.8 | 9.8 | 8.6 | 8.6 | 8.0 | 7.8 | 9.4 |
|  | **9-10** | 11.4 | 10.4 | 8.4 | 8.2 | 8.2 | 8.0 | 9.2 |
|  | **Mean** | **11.9** | **10.2** | **9.0** | **8.4** | **8.0** | **8.0** | **9.9** |
|  | **SE** | **0.4** | **0.1** | **0.1** | **0.1** | **0.1** | **0.2** | **0.2** |
