# Supplementary material for: A novel calcimimetic agent, evocalcet (MT-4580/KHK7580), suppresses the parathyroid cell function with little effect on the gastrointestinal tract or CYP isozymes in vivo and in vitro
Source: PLoS One. 2018 Apr 3;13(4):e0195316. doi: 10.1371/journal.pone.0195316 (PMC5882164; doi:10.1371/journal.pone.0195316)
Supplement: S3 Table — (DOCX) [file pone.0195316.s003.docx]

**S3 Table. The set of raw data for Fig. 4**

(A) Serum PTH levels after the first administration

| **Group** | **Rat** | **Time(h)** | | | | | |
| --- | --- | --- | --- | --- | --- | --- | --- |
|  | **No.** | **0** | **0.5** | **2** | **4** | **8** | **24** |
| **Sham-Vehicle** | **1-1** | 215.689 | 186.925 | 78.079 | 98.663 | 158.912 | 161.181 |
|  | **1-2** | 145.775 | 280.588 | 439.498 | 158.304 | 127.347 | 236.833 |
|  | **1-3** | 118.773 | 216.201 | 195.666 | 165.261 | 149.258 | 69.003 |
|  | **1-4** | 50.310 | 119.328 | 164.727 | 122.823 | 142.068 | 102.593 |
|  | **1-5** | 131.642 | 449.531 | 243.696 | 155.268 | 150.148 | 163.825 |
|  | **1-6** | 174.601 | 645.366 | 204.189 | 330.837 | 139.466 | 101.332 |
|  | **1-7** | 134.591 | 235.556 | 150.966 | 107.834 | 96.192 | 121.642 |
|  | **1-8** | 106.326 | 285.743 | 317.027 | 114.348 | 186.986 | 74.111 |
|  | **1-9** | 144.184 | 537.309 | 283.069 | 199.793 | 215.607 | 170.555 |
|  | **1-10** | 206.516 | 125.505 | 553.401 | 208.521 | 205.953 | 91.297 |
|  | **1-11** | 221.819 | 480.168 | 303.577 | 254.816 | 332.626 | 319.637 |
|  | **1-12** | 156.351 | 96.861 | 91.706 | 129.907 | 160.145 | 97.606 |
|  | **Mean** | **150.548** | **304.923** | **252.133** | **170.531** | **172.059** | **142.468** |
|  | **S.E.** | **14.237** | **52.152** | **40.184** | **19.789** | **17.425** | **21.374** |
| **Nx-vehicle** | **2-1** | 1248.781 | 1321.725 | 376.855 | 555.297 | 270.410 | 1082.015 |
|  | **2-2** | 3167.195 | 4469.437 | 4822.438 | 3925.405 | 3864.626 | 3301.119 |
|  | **2-3** | 2772.234 | 2573.722 | 5219.723 | 1016.040 | 2426.246 | 2064.177 |
|  | **2-4** | 3123.908 | 4223.203 | 3996.927 | 5120.763 | 4561.701 | 4124.020 |
|  | **2-5** | 3660.896 | 3172.608 | 3406.659 | 4738.869 | 5229.955 | 4465.369 |
|  | **2-6** | 2207.403 | 2473.786 | 2228.118 | 1863.843 | 2973.336 | 1619.215 |
|  | **2-7** | 4646.647 | 4740.670 | 4467.234 | 5171.992 | 5006.603 | 4614.204 |
|  | **2-8** | 1556.233 | 1624.938 | 1828.613 | 1259.218 | 1449.838 | 1225.028 |
|  | **2-9** | 3244.877 | 3181.848 | 2896.180 | 2946.920 | 3171.905 | 3193.258 |
|  | **2-10** | 2077.309 | 2760.463 | 2436.083 | 1860.365 | 2128.394 | 2191.567 |
|  | **2-11** | 1970.848 | 3009.939 | 2254.752 | 2990.243 | 1800.071 | 2242.042 |
|  | **2-12** | 3491.025 | 5704.358 | 1984.642 | 3187.569 | 2679.935 | 1855.667 |
|  | **Mean** | **2763.946** | **3271.391** | **2993.185** | **2886.377** | **2963.585** | **2664.807** |
|  | **S.E.** | **282.920** | **373.370** | **411.242** | **464.750** | **432.442** | **358.292** |

| **Group** | **Rat** | **Time(h)** | | | | | |
| --- | --- | --- | --- | --- | --- | --- | --- |
|  | **No.** | **0** | **0.5** | **2** | **4** | **8** | **24** |
| **Nx-0.03 mg/kg** | **3-1** | 2977.436 | 1420.446 | 892.661 | 1116.916 | 958.210 | 2211.997 |
|  | **3-2** | 1709.257 | 434.216 | 957.691 | 872.473 | 1404.989 | 2040.141 |
|  | **3-3** | 736.362 | 55.317 | 668.938 | 149.262 | 437.824 | 1241.853 |
|  | **3-4** | 1492.356 | 322.608 | 397.274 | 347.852 | 978.409 | 1209.705 |
|  | **3-5** | 1948.854 | 2091.549 | 1591.573 | 1126.719 | 1548.095 | 2393.467 |
|  | **3-6** | 2855.064 | 2666.120 | 2567.476 | 4206.392 | 3071.936 | 3649.938 |
|  | **3-7** | 3146.838 | 4101.960 | 4890.509 | 4679.877 | 5964.855 | 4890.842 |
|  | **3-8** | 3130.460 | 5220.639 | 4068.711 | 4835.119 | 4334.746 | 5921.359 |
|  | **3-9** | 2411.903 | 2506.611 | 2185.504 | 2331.859 | 2555.658 | 2295.822 |
|  | **3-10** | 1952.597 | 3090.422 | 3005.600 | 3883.490 | 4291.517 | 3334.469 |
|  | **3-11** | 3363.037 | 3558.006 | 4664.020 | 3137.288 | 2662.474 | 3685.692 |
|  | **3-12** | 1097.629 | 125.411 | 345.560 | 547.391 | 769.912 | 634.649 |
|  | **Mean** | **2235.149** | **2132.775** | **2186.293** | **2269.553** | **2414.885** | **2792.495** |
|  | **S.E.** | **252.290** | **490.836** | **478.633** | **516.733** | **499.196** | **451.383** |
| **Nx-0.1 mg/kg** | **4-1** | 2191.024 | 697.065 | 921.070 | 1346.496 | 1602.872 | 2327.970 |
|  | **4-2** | 4476.799 | 166.990 | 152.688 | 622.337 | 1239.629 | 3050.546 |
|  | **4-3** | 4734.069 | 643.814 | 1274.188 | 1091.618 | 2688.836 | 5188.511 |
|  | **4-4** | 3561.219 | 188.145 | 268.767 | 881.643 | 1069.819 | 1270.996 |
|  | **4-5** | 1608.879 | 87.713 | 48.850 | 178.355 | 616.193 | 1634.838 |
|  | **4-6** | 3394.625 | 1427.254 | 837.618 | 1445.792 | 1780.899 | 3384.643 |
|  | **4-7** | 2344.750 | 2994.134 | 3213.565 | 3885.042 | 3893.446 | 3324.854 |
|  | **4-8** | 2855.532 | 497.193 | 734.412 | 1344.283 | 2481.366 | 2293.418 |
|  | **4-9** | 1478.317 | 52.922 | 45.617 | 585.655 | 541.217 | 169.556 |
|  | **4-10** | 2515.323 | 216.107 | 171.332 | 429.439 | 1134.867 | 1281.211 |
|  | **4-11** | 3938.529 | 4171.956 | 4414.015 | 2676.862 | 4131.208 | 4614.204 |
|  | **4-12** | 2968.311 | 129.788 | 69.629 | 114.300 | 608.661 | 541.810 |
|  | **Mean** | **3005.615** | **939.423** | **1012.646** | **1216.819** | **1815.751** | **2423.546** |
|  | **S.E.** | **303.145** | **380.409** | **402.193** | **315.392** | **356.778** | **447.857** |

| **Group** | **Rat** | **Time(h)** | | | | | |
| --- | --- | --- | --- | --- | --- | --- | --- |
|  | **No.** | **0** | **0.5** | **2** | **4** | **8** | **24** |
| **Nx-0.3 mg/kg** | **5-1** | 1744.120 | 280.057 | 1564.496 | 2169.635 | 3597.115 | 2544.592 |
|  | **5-2** | 1258.608 | 54.240 | 49.749 | 72.144 | 972.589 | 1343.404 |
|  | **5-3** | 4765.291 | 516.402 | 390.394 | 1298.430 | 1732.284 | 2001.985 |
|  | **5-4** | 2135.103 | 147.295 | 84.719 | 583.758 | 821.951 | 166.251 |
|  | **5-5** | 1977.165 | 58.671 | 45.677 | 62.623 | 476.853 | 349.524 |
|  | **5-6** | 2399.034 | 90.767 | 41.605 | 15.500 | 181.397 | 396.995 |
|  | **5-7** | 1632.745 | 63.701 | 53.761 | 101.725 | 928.767 | 1271.297 |
|  | **5-8** | 2446.532 | 724.784 | 598.359 | 865.516 | 2068.823 | 1890.519 |
|  | **5-9** | 2253.029 | 78.911 | 15.500 | 39.329 | 597.706 | 1492.426 |
|  | **5-10** | 2449.574 | 232.642 | 339.790 | 1539.079 | 2494.718 | 2261.571 |
|  | **5-11** | 3238.325 | 100.408 | 59.629 | 94.839 | 942.804 | 426.138 |
|  | **5-12** | 2017.644 | 60.168 | 41.784 | 529.050 | 758.615 | 211.018 |
|  | **Mean** | **2359.764** | **200.671** | **273.789** | **614.302** | **1297.802** | **1196.310** |
|  | **S.E.** | **261.421** | **61.645** | **128.980** | **206.589** | **286.016** | **248.931** |
| **Nx-1 mg/kg** | **6-1** | 3077.112 | 86.815 | 436.337 | 85.737 | 1093.100 | 1397.785 |
|  | **6-2** | 1497.503 | 55.617 | 62.922 | 58.910 | 39.329 | 162.646 |
|  | **6-3** | 1942.068 | 58.671 | 454.093 | 65.617 | 569.290 | 843.460 |
|  | **6-4** | 2386.399 | 124.925 | 657.175 | 337.417 | 1479.623 | 1084.118 |
|  | **6-5** | 1914.692 | 59.749 | 75.318 | 46.036 | 232.066 | 494.640 |
|  | **6-6** | 2968.311 | 120.305 | 320.258 | 510.077 | 828.114 | 308.613 |
|  | **6-7** | 2088.774 | 69.150 | 138.484 | 70.827 | 246.103 | 343.515 |
|  | **6-8** | 3335.895 | 90.707 | 294.956 | 69.928 | 300.196 | 290.336 |
|  | **6-9** | 2674.664 | 86.036 | 634.314 | 71.425 | 164.279 | 534.299 |
|  | **6-10** | 515.015 | 15.500 | 15.500 | 15.500 | 73.581 | 254.583 |
|  | **6-11** | 3504.128 | 119.089 | 2124.913 | 389.594 | 968.481 | 1418.215 |
|  | **6-12** | 3954.764 | 145.593 | 2858.893 | 1906.218 | 3469.757 | 3238.025 |
|  | **Mean** | **2488.277** | **86.013** | **672.764** | **302.274** | **788.660** | **864.186** |
|  | **S.E.** | **277.784** | **10.651** | **256.825** | **153.137** | **277.241** | **250.663** |

(B) Serum Ca levels after the first administration

| **Group** | **Rat** | **Time(h)** | | | | | |
| --- | --- | --- | --- | --- | --- | --- | --- |
|  | **No.** | **0** | **0.5** | **2** | **4** | **8** | **24** |
| **Sham-Vehicle** | **1-1** | 8.878 | 7.709 | 7.953 | 8.666 | 9.025 | 8.424 |
|  | **1-2** | 9.080 | 8.239 | 8.274 | 8.872 | 8.765 | 8.823 |
|  | **1-3** | 8.293 | 8.104 | 7.706 | 8.333 | 8.685 | 8.184 |
|  | **1-4** | 8.720 | 8.038 | 7.925 | 8.695 | 8.524 | 8.933 |
|  | **1-5** | 8.566 | 8.035 | 8.326 | 8.660 | 9.080 | 8.390 |
|  | **1-6** | 8.676 | 7.762 | 8.058 | 8.609 | 8.351 | 8.481 |
|  | **1-7** | 9.182 | 8.268 | 8.036 | 8.406 | 8.919 | 8.668 |
|  | **1-8** | 8.744 | 8.117 | 7.647 | 8.199 | 8.431 | 8.841 |
|  | **1-9** | 8.686 | 8.166 | 8.357 | 8.514 | 8.948 | 8.914 |
|  | **1-10** | 9.764 | 8.762 | 8.900 | 9.149 | 9.420 | 9.224 |
|  | **1-11** | 8.587 | 8.555 | 8.141 | 9.031 | 9.141 | 8.788 |
|  | **1-12** | 9.124 | 8.726 | 8.286 | 8.968 | 8.813 | 8.895 |
|  | **Mean** | **8.858** | **8.207** | **8.134** | **8.675** | **8.842** | **8.714** |
|  | **S.E.** | **0.111** | **0.096** | **0.096** | **0.084** | **0.090** | **0.084** |
| **Nx-Vehicle** | **2-1** | 8.946 | 7.423 | 8.039 | 8.355 | 8.486 | 8.168 |
|  | **2-2** | 7.321 | 6.952 | 7.320 | 7.491 | 7.705 | 7.381 |
|  | **2-3** | 8.251 | 7.584 | 7.752 | 8.663 | 8.434 | 8.023 |
|  | **2-4** | 7.160 | 7.084 | 6.539 | 7.326 | 7.423 | 7.423 |
|  | **2-5** | 7.369 | 6.478 | 6.320 | 6.685 | 7.297 | 6.395 |
|  | **2-6** | 7.752 | 7.271 | 7.400 | 7.682 | 8.335 | 7.170 |
|  | **2-7** | 6.848 | 6.379 | 6.202 | 6.802 | 7.015 | 6.854 |
|  | **2-8** | 8.549 | 7.340 | 7.700 | 8.171 | 8.177 | 8.522 |
|  | **2-9** | 8.070 | 7.475 | 7.496 | 7.860 | 7.680 | 7.969 |
|  | **2-10** | 7.622 | 7.189 | 6.965 | 8.066 | 7.541 | 7.716 |
|  | **2-11** | 8.399 | 7.182 | 7.277 | 7.958 | 7.673 | 7.919 |
|  | **2-12** | 7.813 | 7.301 | 7.545 | 8.171 | 8.200 | 6.838 |
|  | **Mean** | **7.842** | **7.138** | **7.213** | **7.769** | **7.831** | **7.532** |
|  | **S.E.** | **0.180** | **0.108** | **0.169** | **0.174** | **0.139** | **0.183** |

| **Group** | **Rat** | **Time(h)** | | | | | |
| --- | --- | --- | --- | --- | --- | --- | --- |
|  | **No.** | **0** | **0.5** | **2** | **4** | **8** | **24** |
| **Nx-0.03 mg/kg** | **3-1** | 8.077 | 7.189 | 7.311 | 7.885 | 8.258 | 7.385 |
|  | **3-2** | 8.881 | 7.821 | 7.468 | 7.869 | 7.943 | 7.805 |
|  | **3-3** | 8.577 | 7.887 | 7.761 | 7.822 | 7.943 | 8.029 |
|  | **3-4** | 8.645 | 7.354 | 7.570 | 8.203 | 8.091 | 8.093 |
|  | **3-5** | 8.453 | 6.982 | 7.085 | 7.761 | 7.712 | 7.647 |
|  | **3-6** | 8.169 | 7.350 | 7.002 | 7.799 | 8.264 | 7.290 |
|  | **3-7** | 6.475 | 5.843 | 6.221 | 6.129 | 7.265 | 7.337 |
|  | **3-8** | 7.379 | 6.570 | 6.752 | 7.183 | 7.625 | 7.644 |
|  | **3-9** | 7.410 | 6.547 | 6.943 | 6.895 | 7.368 | 7.429 |
|  | **3-10** | 6.746 | 6.666 | 6.746 | 7.539 | 8.097 | 7.568 |
|  | **3-11** | 6.842 | 6.639 | 6.851 | 6.860 | 7.474 | 7.040 |
|  | **3-12** | 8.689 | 7.943 | 7.851 | 8.183 | 8.633 | 8.620 |
|  | **Mean** | **7.862** | **7.066** | **7.130** | **7.511** | **7.889** | **7.657** |
|  | **S.E.** | **0.246** | **0.186** | **0.138** | **0.180** | **0.119** | **0.124** |
| **Nx-0.1 mg/kg** | **4-1** | 7.331 | 6.761 | 6.582 | 7.060 | 7.692 | 7.618 |
|  | **4-2** | 8.275 | 6.580 | 6.412 | 7.085 | 7.609 | 8.042 |
|  | **4-3** | 7.642 | 6.840 | 6.604 | 7.028 | 7.307 | 7.976 |
|  | **4-4** | 7.659 | 7.344 | 7.150 | 7.482 | 7.085 | 7.739 |
|  | **4-5** | 8.173 | 6.409 | 6.977 | 7.364 | 7.975 | 7.539 |
|  | **4-6** | 8.364 | 6.926 | 6.857 | 7.406 | 4.442 | 7.979 |
|  | **4-7** | 6.513 | 6.241 | 5.795 | 5.948 | 6.559 | 6.079 |
|  | **4-8** | 7.933 | 6.919 | 6.903 | 7.326 | 7.908 | 7.486 |
|  | **4-9** | 8.070 | 6.445 | 7.147 | 7.361 | 8.235 | 8.194 |
|  | **4-10** | 7.690 | 6.968 | 6.715 | 7.050 | 7.683 | 7.356 |
|  | **4-11** | 6.674 | 6.307 | 6.267 | 6.742 | 7.310 | 7.483 |
|  | **4-12** | 8.180 | 7.321 | 6.922 | 7.336 | 7.609 | 8.020 |
|  | **Mean** | **7.709** | **6.755** | **6.694** | **7.099** | **7.285** | **7.626** |
|  | **S.E.** | **0.174** | **0.106** | **0.114** | **0.122** | **0.288** | **0.161** |

| **Group** | **Rat** | **Time(h)** | | | | | |
| --- | --- | --- | --- | --- | --- | --- | --- |
|  | **No.** | **0** | **0.5** | **2** | **4** | **8** | **24** |
| **Nx-0.3 mg/kg** | **5-1** | 7.006 | 5.889 | 5.378 | 6.266 | 6.979 | 6.952 |
|  | **5-2** | 8.419 | 7.479 | 6.610 | 7.368 | 8.364 | 8.014 |
|  | **5-3** | 8.245 | 6.978 | 5.851 | 6.374 | 7.034 | 6.996 |
|  | **5-4** | 7.878 | 7.377 | 6.770 | 6.777 | 7.497 | 6.806 |
|  | **5-5** | 7.711 | 6.840 | 6.261 | 7.117 | 7.631 | 7.103 |
|  | **5-6** | 8.450 | 6.455 | 6.505 | 6.974 | 6.623 | 6.740 |
|  | **5-7** | 7.714 | 7.149 | 6.548 | 6.983 | 7.763 | 7.394 |
|  | **5-8** | 7.098 | 5.635 | 5.542 | 6.066 | 6.562 | 6.200 |
|  | **5-9** | 4.207 | 6.564 | 6.131 | 6.980 | 7.172 | 7.223 |
|  | **5-10** | 6.520 | 6.448 | 5.443 | 6.164 | 6.938 | 6.882 |
|  | **5-11** | 8.019 | 7.126 | 6.363 | 6.920 | 6.880 | 7.062 |
|  | **5-12** | 4.857 | 7.390 | 6.233 | 6.577 | 7.169 | 7.837 |
|  | **Mean** | **7.177** | **6.778** | **6.136** | **6.714** | **7.218** | **7.101** |
|  | **S.E.** | **0.396** | **0.172** | **0.138** | **0.120** | **0.149** | **0.140** |
| **Nx-1 mg/kg** | **6-1** | 7.396 | 6.060 | 4.992 | 5.818 | 6.186 | 6.377 |
|  | **6-2** | 7.591 | 6.949 | 6.922 | 5.774 | 6.340 | 5.953 |
|  | **6-3** | 7.831 | 6.468 | 5.230 | 5.723 | 5.987 | 6.177 |
|  | **6-4** | 7.728 | 6.244 | 4.739 | 5.355 | 6.006 | 5.615 |
|  | **6-5** | 8.433 | 7.228 | 5.718 | 6.358 | 6.552 | 6.421 |
|  | **6-6** | 7.868 | 6.211 | 5.002 | 6.431 | 7.439 | 6.566 |
|  | **6-7** | 8.231 | 6.837 | 5.539 | 6.126 | 6.392 | 6.604 |
|  | **6-8** | 7.824 | 6.741 | 5.196 | 5.799 | 6.029 | 5.372 |
|  | **6-9** | 8.217 | 6.643 | 5.119 | 6.025 | 6.067 | 5.928 |
|  | **6-10** | 9.292 | 7.367 | 6.289 | 6.574 | 7.153 | 7.552 |
|  | **6-11** | 6.900 | 5.471 | 4.452 | 5.161 | 5.515 | 5.981 |
|  | **6-12** | 6.102 | 5.306 | 4.286 | 5.301 | 5.916 | 6.493 |
|  | **Mean** | **7.784** | **6.460** | **5.290** | **5.870** | **6.299** | **6.253** |
|  | **S.E.** | **0.229** | **0.184** | **0.216** | **0.131** | **0.155** | **0.162** |

(C) Serum PTH levels before (day 0) and 24 h after evocalcet administration (days 7 and 14)

| **Group** | **Rat**  **No.** | **Days** | | |
| --- | --- | --- | --- | --- |
|  |  | **0 (Before)** | **7 (24 h)** | **14 (24 h)** |
| **Sham-Vehicle** | **1-1** | 215.689 | 111.689 | 84.310 |
|  | **1-2** | 145.775 | 292.328 | 217.202 |
|  | **1-3** | 118.773 | 184.912 | 83.880 |
|  | **1-4** | 50.310 | 84.122 | 91.779 |
|  | **1-5** | 131.642 | 340.238 | 110.104 |
|  | **1-6** | 174.601 | 94.592 | 164.486 |
|  | **1-7** | 134.591 | 263.701 | 318.013 |
|  | **1-8** | 106.326 | 133.556 | 89.952 |
|  | **1-9** | 144.184 | 340.768 | 222.468 |
|  | **1-10** | 206.516 | 216.520 | 151.696 |
|  | **1-11** | 221.819 | 148.267 | 244.339 |
|  | **1-12** | 156.351 | 57.086 | 143.044 |
|  | **Mean** | **150.548** | **188.982** | **160.106** |
|  | **S.E.** | **14.237** | **28.993** | **21.882** |
| **Nx-Vehicle** | **2-1** | 1248.781 | 818.203 | 853.058 |
|  | **2-2** | 3167.195 | 4271.380 | 4690.930 |
|  | **2-3** | 2772.234 | 838.083 | 1303.376 |
|  | **2-4** | 3123.908 | 4058.149 | 3649.137 |
|  | **2-5** | 3660.896 | 3684.580 | 3160.803 |
|  | **2-6** | 2207.403 | 1957.636 | 1652.937 |
|  | **2-7** | 4646.647 | 4954.051 | 4948.370 |
|  | **2-8** | 1556.233 | 1210.825 | 1065.858 |
|  | **2-9** | 3244.877 | 4720.984 | 4699.351 |
|  | **2-10** | 2077.309 | 1592.183 | 1844.779 |
|  | **2-11** | 1970.848 | 2357.879 | 2396.929 |
|  | **2-12** | 3491.025 | 3573.832 | 4771.530 |
|  | **Mean** | **2763.946** | **2836.482** | **2919.755** |
|  | **S.E.** | **282.920** | **444.745** | **458.861** |

| **Group** | **Rat**  **No.** | **Days** | | |
| --- | --- | --- | --- | --- |
|  |  | **0 (Before)** | **7 (24 h)** | **14 (24 h)** |
| **Nx-0.03 mg/kg** | **3-1** | 2977.436 | 170.127 | 465.344 |
|  | **3-2** | 1709.257 | 208.561 | 1621.501 |
|  | **3-3** | 736.362 | 3498.638 | 608.285 |
|  | **3-4** | 1492.356 | 332.146 | 1702.644 |
|  | **3-5** | 1948.854 | 1090.222 | 1919.474 |
|  | **3-6** | 2855.064 | 2341.644 | 2965.469 |
|  | **3-7** | 3146.838 | 175.760 | 4997.693 |
|  | **3-8** | 3130.460 | 1003.083 | 3995.599 |
|  | **3-9** | 2411.903 | 756.245 | 1879.977 |
|  | **3-10** | 1952.597 | 2355.229 | 3492.362 |
|  | **3-11** | 3363.037 | 3361.800 | 3523.261 |
|  | **3-12** | 1097.629 | 1990.106 | 468.569 |
|  | **Mean** | **2235.149** | **1440.297** | **2303.348** |
|  | **S.E.** | **252.290** | **354.836** | **428.474** |
| **Nx-0.1 mg/kg** | **4-1** | 2191.024 | 1659.111 | 1994.437 |
|  | **4-2** | 4476.799 | 1709.141 | 1611.022 |
|  | **4-3** | 4734.069 | 1368.206 | 2757.774 |
|  | **4-4** | 3561.219 | 692.961 | 1453.572 |
|  | **4-5** | 1608.879 | 2049.745 | 176.238 |
|  | **4-6** | 3394.625 | 3311.770 | 2214.760 |
|  | **4-7** | 2344.750 | 3632.825 | 1548.955 |
|  | **4-8** | 2855.532 | 4438.329 | 1921.086 |
|  | **4-9** | 1478.317 | 2720.020 | 242.603 |
|  | **4-10** | 2515.323 | 2668.995 | 1372.697 |
|  | **4-11** | 3938.529 | 3977.154 | 1155.599 |
|  | **4-12** | 2968.311 | 674.076 | 291.504 |
|  | **Mean** | **3005.615** | **2408.528** | **1395.021** |
|  | **S.E.** | **303.145** | **361.746** | **235.616** |

| **Group** | **Rat**  **No.** | **Days** | | |
| --- | --- | --- | --- | --- |
|  |  | **0 (Before)** | **7 (24 h)** | **14 (24 h)** |
| **Nx-0.3 mg/kg** | **5-1** | 1744.120 | 272.176 | 2259.361 |
|  | **5-2** | 1258.608 | 15.500 | 243.410 |
|  | **5-3** | 4765.291 | 823.173 | 255.500 |
|  | **5-4** | 2135.103 | 404.707 | 180.000 |
|  | **5-5** | 1977.165 | 530.280 | 101.543 |
|  | **5-6** | 2399.034 | 15.500 | 985.789 |
|  | **5-7** | 1632.745 | 516.033 | 134.323 |
|  | **5-8** | 2446.532 | 370.580 | 160.654 |
|  | **5-9** | 2253.029 | 777.450 | 218.153 |
|  | **5-10** | 2449.574 | 52.037 | 43.180 |
|  | **5-11** | 3238.325 | 15.500 | 221.109 |
|  | **5-12** | 2017.644 | 862.269 | 481.197 |
|  | **Mean** | **2359.764** | **387.934** | **440.352** |
|  | **S.E.** | **261.421** | **93.166** | **180.289** |
| **Nx-1 mg/kg** | **6-1** | 3077.112 | 740.010 | 671.158 |
|  | **6-2** | 1497.503 | 15.500 | 155.818 |
|  | **6-3** | 1942.068 | 123.742 | 37.909 |
|  | **6-4** | 2386.399 | 15.500 | 236.155 |
|  | **6-5** | 1914.692 | 15.500 | 140.234 |
|  | **6-6** | 2968.311 | 91.603 | 473.942 |
|  | **6-7** | 2088.774 | 15.500 | 170.596 |
|  | **6-8** | 3335.895 | 1068.686 | 576.580 |
|  | **6-9** | 2674.664 | 15.500 | 15.500 |
|  | **6-10** | 515.015 | 15.500 | 15.500 |
|  | **6-11** | 3504.128 | 1281.729 | 580.073 |
|  | **6-12** | 3954.764 | 4297.827 | 4058.155 |
|  | **Mean** | **2488.277** | **641.383** | **594.302** |
|  | **S.E.** | **277.784** | **357.734** | **322.220** |

(D) Serum Ca levels before (day 0) and 24 h after evocalcet administration (days 7 and 14)

| **Group** | **Rat**  **No.** | **Days** | | |
| --- | --- | --- | --- | --- |
|  |  | **0 (Before)** | **7 (24 h)** | **14 (24 h)** |
| **Sham-Vehicle** | **1-1** | 8.878 | 8.623 | 8.871 |
|  | **1-2** | 9.080 | 8.736 | 8.453 |
|  | **1-3** | 8.293 | 8.922 | 8.846 |
|  | **1-4** | 8.720 | 8.878 | 8.548 |
|  | **1-5** | 8.566 | 8.542 | 8.809 |
|  | **1-6** | 8.676 | 8.711 | 9.044 |
|  | **1-7** | 9.182 | 9.104 | 9.117 |
|  | **1-8** | 8.744 | 8.661 | 8.875 |
|  | **1-9** | 8.686 | 9.000 | 8.846 |
|  | **1-10** | 9.764 | 11.307 | 10.183 |
|  | **1-11** | 8.587 | 8.601 | 8.632 |
|  | **1-12** | 9.124 | 9.072 | 9.057 |
|  | **Mean** | **8.858** | **9.013** | **8.940** |
|  | **S.E.** | **0.111** | **0.216** | **0.127** |
| **Nx-Vehicle** | **2-1** | 8.946 | 8.225 | 8.114 |
|  | **2-2** | 7.321 | 7.794 | 7.878 |
|  | **2-3** | 8.251 | 8.582 | 7.881 |
|  | **2-4** | 7.160 | 6.165 | 7.104 |
|  | **2-5** | 7.369 | 6.510 | 7.233 |
|  | **2-6** | 7.752 | 7.977 | 8.425 |
|  | **2-7** | 6.848 | 6.514 | 5.551 |
|  | **2-8** | 8.549 | 8.410 | 8.283 |
|  | **2-9** | 8.070 | 7.223 | 6.010 |
|  | **2-10** | 7.622 | 7.964 | 8.233 |
|  | **2-11** | 8.399 | 8.049 | 7.956 |
|  | **2-12** | 7.813 | 7.757 | 7.334 |
|  | **Mean** | **7.842** | **7.598** | **7.500** |
|  | **S.E.** | **0.180** | **0.232** | **0.263** |

| **Group** | **Rat**  **No.** | **Days** | | |
| --- | --- | --- | --- | --- |
|  |  | **0 (Before)** | **7 (24 h)** | **14 (24 h)** |
| **Nx-0.03 mg/kg** | **3-1** | 8.077 | 6.316 | 7.991 |
|  | **3-2** | 8.881 | 6.947 | 8.107 |
|  | **3-3** | 8.577 | 6.611 | 7.192 |
|  | **3-4** | 8.645 | 7.154 | 7.532 |
|  | **3-5** | 8.453 | 6.338 | 7.865 |
|  | **3-6** | 8.169 | 6.972 | 7.809 |
|  | **3-7** | 6.475 | 4.756 | 6.629 |
|  | **3-8** | 7.379 | 6.463 | 7.365 |
|  | **3-9** | 7.410 | 7.204 | 7.620 |
|  | **3-10** | 6.746 | 6.649 | 6.148 |
|  | **3-11** | 6.842 | 7.424 | 7.312 |
|  | **3-12** | 8.689 | 7.568 | 8.717 |
|  | **Mean** | **7.862** | **6.700** | **7.524** |
|  | **S.E.** | **0.246** | **0.213** | **0.196** |
| **Nx-0.1 mg/kg** | **4-1** | 7.331 | 7.427 | 7.048 |
|  | **4-2** | 8.275 | 8.099 | 7.642 |
|  | **4-3** | 7.642 | 7.493 | 6.969 |
|  | **4-4** | 7.659 | 7.242 | 7.601 |
|  | **4-5** | 8.173 | 7.637 | 6.617 |
|  | **4-6** | 8.364 | 7.025 | 6.853 |
|  | **4-7** | 6.513 | 6.448 | 5.928 |
|  | **4-8** | 7.933 | 7.138 | 7.145 |
|  | **4-9** | 8.070 | 7.123 | 7.117 |
|  | **4-10** | 7.690 | 6.680 | 6.576 |
|  | **4-11** | 6.674 | 7.302 | 7.375 |
|  | **4-12** | 8.180 | 8.184 | 7.261 |
|  | **Mean** | **7.709** | **7.317** | **7.011** |
|  | **S.E.** | **0.174** | **0.146** | **0.138** |

| **Group** | **Rat**  **No.** | **Days** | | |
| --- | --- | --- | --- | --- |
|  |  | **0 (Before)** | **7 (24 h)** | **14 (24 h)** |
| **Nx-0.3 mg/kg** | **5-1** | 7.006 | 6.297 | 6.733 |
|  | **5-2** | 8.419 | 6.366 | 6.057 |
|  | **5-3** | 8.245 | 7.085 | 5.865 |
|  | **5-4** | 7.878 | 5.154 | 5.887 |
|  | **5-5** | 7.711 | 4.887 | 5.547 |
|  | **5-6** | 8.450 | 5.195 | 5.661 |
|  | **5-7** | 7.714 | 6.617 | 6.601 |
|  | **5-8** | 7.098 | 4.991 | 5.324 |
|  | **5-9** | 4.207 | 5.484 | 6.337 |
|  | **5-10** | 6.520 | 4.834 | 4.673 |
|  | **5-11** | 8.019 | 5.302 | 5.104 |
|  | **5-12** | 4.857 | 6.718 | 7.592 |
|  | **Mean** | **7.177** | **5.744** | **5.948** |
|  | **S.E.** | **0.396** | **0.234** | **0.228** |
| **Nx-1 mg/kg** | **6-1** | 7.396 | 5.540 | 5.359 |
|  | **6-2** | 7.591 | 3.798 | 3.730 |
|  | **6-3** | 7.831 | 5.148 | 4.903 |
|  | **6-4** | 7.728 | 4.511 | 4.736 |
|  | **6-5** | 8.433 | 4.963 | 4.928 |
|  | **6-6** | 7.868 | 5.487 | 5.192 |
|  | **6-7** | 8.231 | 4.665 | 4.708 |
|  | **6-8** | 7.824 | 5.044 | 5.906 |
|  | **6-9** | 8.217 | 4.307 | 3.598 |
|  | **6-10** | 9.292 | 5.493 | 4.859 |
|  | **6-11** | 6.900 | 4.570 | 4.220 |
|  | **6-12** | 6.102 | 6.793 | 5.475 |
|  | **Mean** | **7.784** | **5.027** | **4.801** |
|  | **S.E.** | **0.229** | **0.221** | **0.197** |
