# Supplementary material for: A novel calcimimetic agent, evocalcet (MT-4580/KHK7580), suppresses the parathyroid cell function with little effect on the gastrointestinal tract or CYP isozymes in vivo and in vitro
Source: PLoS One. 2018 Apr 3;13(4):e0195316. doi: 10.1371/journal.pone.0195316 (PMC5882164; doi:10.1371/journal.pone.0195316)
Supplement: S4 Table — (DOCX) [file pone.0195316.s004.docx]

**S4 Table. The set of raw data for Fig. 5**

(A) Evocalcet

| **Group** | **Rat No.** | **Gastric Emptying (%)** |
| --- | --- | --- |
| **Vehicle** | 2-1 | 43.09 |
|  | 2-2 | 75.49 |
|  | 2-3 | 78.82 |
|  | 2-4 | 70.92 |
|  | 2-5 | 44.76 |
|  | 2-6 | 66.77 |
|  | 2-7 | 78.40 |
|  | 2-8 | 65.73 |
|  | **mean** | **65.50** |
|  | **SE** | **5.01** |
| **0.3 mg/kg** | 3-1 | 75.29 |
|  | 3-2 | 74.04 |
|  | 3-3 | 68.43 |
|  | 3-4 | 31.88 |
|  | 3-5 | 68.64 |
|  | 3-6 | 79.44 |
|  | 3-7 | 65.73 |
|  | 3-8 | 79.23 |
|  | **mean** | **67.83** |
|  | **SE** | **5.44** |
| **1 mg/kg** | 4-1 | 38.94 |
|  | 4-2 | 71.96 |
|  | 4-3 | 52.65 |
|  | 4-4 | 74.45 |
|  | 4-5 | 73.42 |
|  | 4-6 | 65.94 |
|  | 4-7 | 77.15 |
|  | 4-8 | 72.59 |
|  | **mean** | **65.89** |
|  | **SE** | **4.71** |
| **Group** | **Rat No.** | **Gastric Emptying (%)** |
| **3 mg/kg** | 5-1 | 69.47 |
|  | 5-2 | 67.19 |
|  | 5-3 | 53.48 |
|  | 5-4 | 63.03 |
|  | 5-5 | 78.82 |
|  | 5-6 | 61.16 |
|  | 5-7 | 64.49 |
|  | 5-8 | 34.99 |
|  | **mean** | **61.58** |
|  | **SE** | **4.58** |

(B) Cinacalcet

| **Group** | **Rat No.** | **Gastric Emptying (%)** |
| --- | --- | --- |
| **Vehicle** | 2-1 | 43.09 |
|  | 2-2 | 75.49 |
|  | 2-3 | 78.82 |
|  | 2-4 | 70.92 |
|  | 2-5 | 44.76 |
|  | 2-6 | 66.77 |
|  | 2-7 | 78.40 |
|  | 2-8 | 65.73 |
|  | **mean** | **65.50** |
|  | **SE** | **5.01** |
| **10 mg/kg** | 6-1 | 44.96 |
|  | 6-2 | 61.79 |
|  | 6-3 | 57.42 |
|  | 6-4 | 52.23 |
|  | 6-5 | 44.34 |
|  | 6-6 | 52.86 |
|  | 6-7 | 67.60 |
|  | 6-8 | 60.12 |
|  | **mean** | **55.17** |
|  | **SE** | **2.88** |
| **30 mg/kg** | 7-1 | 28.35 |
|  | 7-2 | 14.64 |
|  | 7-3 | 23.99 |
|  | 7-4 | 37.69 |
|  | 7-5 | 38.94 |
|  | 7-6 | 32.71 |
|  | 7-7 | 40.60 |
|  | 7-8 | 68.85 |
|  | **mean** | **35.72** |
|  | **SE** | **5.64** |

| **Group** | **Rat No.** | **Gastric Emptying (%)** |
| --- | --- | --- |
| **100 mg/kg** | 8-1 | 20.46 |
|  | 8-2 | 31.67 |
|  | 8-3 | 44.76 |
|  | 8-4 | 1.77 |
|  | 8-5 | 30.22 |
|  | 8-6 | 22.74 |
|  | 8-7 | 1.35 |
|  | 8-8 | 7.17 |
|  | **mean** | **20.02** |
|  | **SE** | **5.52** |
