# Supplementary material for: A novel calcimimetic agent, evocalcet (MT-4580/KHK7580), suppresses the parathyroid cell function with little effect on the gastrointestinal tract or CYP isozymes in vivo and in vitro
Source: PLoS One. 2018 Apr 3;13(4):e0195316. doi: 10.1371/journal.pone.0195316 (PMC5882164; doi:10.1371/journal.pone.0195316)
Supplement: S5 Table — (DOCX) [file pone.0195316.s005.docx]

**S5 Table. The set of raw data for Table 1**

| Dose (mg/kg) | Animal No. | C_max_ (ng/mL) | t_max_  (h) | AUC_0-∞_ (ng·h/mL) | t_1/2_  (h) | F  (%) |
| --- | --- | --- | --- | --- | --- | --- |
| 0.1 | 01101 | 220.8 | 0.25 | 981 | 5.71 | 97.8 |
|  | 01102 | 243.3 | 0.25 | 1099 | 7.00 | 109.5 |
|  | 01103 | 228.7 | 0.25 | 637 | 4.72 | 63.5 |
|  | 01104 | 196.1 | 0.25 | 557 | 9.22 | 55.5 |
|  | **Mean** | **222.2** | **0.25** | **819** | **6.66** | **81.6** |
|  | **S.D.** | **19.8** | **0.00** | **262** | **1.94** | **26.1** |
| 0.3 | 02106 | 531.0 | 0.50 | 2986 | 5.78 | 99.2 |
|  | 02107 | 570.5 | 0.25 | 2260 | 5.68 | 75.1 |
|  | 02108 | 662.3 | 0.25 | 2205 | 6.20 | 73.2 |
|  | 02109 | 634.7 | 0.50 | 2861 | 5.41 | 95.0 |
|  | **Mean** | **599.6** | **0.38** | **2578** | **5.77** | **85.6** |
|  | **S.D.** | **59.8** | **0.14** | **403** | **0.33** | **13.4** |
| 1 | 03111 | 1806 | 1.00 | 7892 | 6.99 | 78.7 |
|  | 03112 | 2303 | 0.25 | 7970 | 5.92 | 79.4 |
|  | 03113 | 2493 | 1.00 | 7815 | 5.56 | 77.9 |
|  | 03114 | 2151 | 1.00 | 10104 | 6.05 | 100.7 |
|  | **Mean** | **2188** | **0.81** | **8445** | **6.13** | **84.2** |
|  | **S.D.** | **291** | **0.38** | **1108** | **0.61** | **11.0** |
