# Supplementary material for: A novel calcimimetic agent, evocalcet (MT-4580/KHK7580), suppresses the parathyroid cell function with little effect on the gastrointestinal tract or CYP isozymes in vivo and in vitro
Source: PLoS One. 2018 Apr 3;13(4):e0195316. doi: 10.1371/journal.pone.0195316 (PMC5882164; doi:10.1371/journal.pone.0195316)
Supplement: S6 Table — (DOCX) [file pone.0195316.s006.docx]

**S6 Table. The set of raw data for Table 2**

|  | **Plasma concentration (ng/mL)** | | | | |
| --- | --- | --- | --- | --- | --- |
| **Dose**  **(mg/kg)** | **Day 1** | | **Day 7** | **Day 14** | |
|  | **0.5 h** | **24 h** | **24 h** | **0.5 h** | **24 h** |
| 0.03 | 71.01 | 8.351 | 8.121 | 33.49 | 23.20 |
|  | 54.36 | 3.032 | 3.188 | 25.01 | 2.138 |
|  | 55.10 | 4.071 | 1.786 | 68.07 | 14.63 |
|  | 69.80 | 7.607 | 7.178 | 72.29 | 13.95 |
|  | 73.84 | 2.316 | 2.139 | 29.04 | 5.827 |
|  | 36.58 | 1.641 | 2.975 | 19.34 | 3.012 |
|  | 70.85 | 1.374 | 2.144 | 56.51 | 3.971 |
|  | 82.54 | 5.810 | 14.97 | 53.07 | 9.010 |
|  | 69.68 | 8.969 | 24.57 | 90.16 | 15.92 |
|  | 77.69 | 1.766 | 7.857 | 75.39 | 10.68 |
|  | 41.93 | 7.813 | 2.119 | 23.11 | 0.6289 |
|  | 76.13 | 3.603 | 5.014 | 72.02 | 6.008 |
| **Mean** | **64.96** | **4.696** | **6.838** | **51.46** | **9.081** |
| **S.D.** | **14.58** | **2.861** | **6.788** | **24.48** | **6.781** |
| 0.1 | 202.3 | 15.16 | 58.71 | 127.4 | 5.812 |
|  | 254.1 | 12.10 | 48.75 | 39.41 | 5.988 |
|  | 209.7 | 8.688 | 30.51 | 36.70 | 9.558 |
|  | 142.8 | 14.16 | 37.24 | 54.83 | 3.499 |
|  | 254.2 | 21.79 | 78.95 | 93.73 | 32.22 |
|  | 143.9 | 16.89 | 69.59 | 108.2 | 49.20 |
|  | 56.83 | 67.75 | 161.4 | 128.5 | 104.7 |
|  | 269.5 | 32.61 | 61.03 | 174.0 | 11.96 |
|  | 203.1 | 25.17 | 63.89 | 174.2 | 38.92 |
|  | 268.0 | 26.87 | 23.11 | 247.3 | 48.85 |
|  | 192.0 | 5.428 | 3.518 | 190.0 | 5.879 |
|  | 254.5 | 14.79 | 4.764 | 231.5 | 34.30 |
| **Mean** | **204.2** | **21.78** | **53.46** | **133.8** | **29.24** |
| **S.D.** | **64.1** | **16.45** | **41.95** | **71.2** | **29.53** |

|  | **Plasma concentration (ng/mL)** | | | | |
| --- | --- | --- | --- | --- | --- |
| **Dose**  **(mg/kg)** | **Day 1** | | **Day 7** | **Day 14** | |
|  | **0.5 h** | **24 h** | **24 h** | **0.5 h** | **24 h** |
| 0.3 | 615.2 | 47.41 | 144.2 | 657.8 | 25.43 |
|  | 628.1 | 10.19 | 262.8 | 465.0 | 27.06 |
|  | 301.2 | 81.90 | 81.84 | 436.6 | 16.7 |
|  | 201.8 | 150.0 | 143.4 | 239.9 | 49.13 |
|  | 693.2 | 110.1 | 145.9 | 939.6 | 76.15 |
|  | 546.9 | 148.6 | 248.2 | 857.2 | 69.19 |
|  | 428.9 | 24.07 | 70.42 | 442.3 | 41.49 |
|  | 644.0 | 229.3 | 247.3 | 806.0 | 220.1 |
|  | 678.4 | 53.95 | 325.0 | 866.7 | 319.7 |
|  | 635.9 | 78.49 | 343.7 | 933.7 | 63.13 |
|  | 572.4 | 128.0 | 260.3 | 1279 | 104.4 |
|  | 599.6 | 23.71 | 14.20 | 318.9 | 12.44 |
| **Mean** | **545.5** | **90.48** | **190.6** | **686.9** | **102.1** |
| **S.D.** | **154.9** | **65.16** | **105.3** | **310.2** | **97.1** |
| 1 | 1329 | 258.6 | 75.60 | 514.8 | 85.74 |
|  | 708.1 | 177.3 | 1147 | 2690 | 848.2 |
|  | 817.9 | 175.7 | 341.4 | 556.0 | 519.8 |
|  | 1630 | 451.5 | 324.5 | 3561 | 797.9 |
|  | 1050 | 289.0 | 1562 | 1432 | 354.0 |
|  | 2575 | 200.6 | 109.1 | 1426 | 227.4 |
|  | 3158 | 270.2 | 414.6 | 1082 | 149.7 |
|  | 2107 | 775.8 | 610.7 | 1539 | 248.4 |
|  | 1568 | 479.9 | 476.0 | 3527 | 887.8 |
|  | 1003 | 93.46 | 827.3 | 2325 | 198.7 |
|  | 1691 | 159.4 | 776.0 | 2235 | 1382 |
|  | 1980 | 96.97 | 22.24 | 335.6 | 188.0 |
| **Mean** | **1635** | **285.7** | **557.2** | **1769** | **490.6** |
| **S.D.** | **733** | **196.8** | **460.0** | **1109** | **402.2** |
