# Supplementary material for: A novel calcimimetic agent, evocalcet (MT-4580/KHK7580), suppresses the parathyroid cell function with little effect on the gastrointestinal tract or CYP isozymes in vivo and in vitro
Source: PLoS One. 2018 Apr 3;13(4):e0195316. doi: 10.1371/journal.pone.0195316 (PMC5882164; doi:10.1371/journal.pone.0195316)
Supplement: S7 Table — (DOCX) [file pone.0195316.s007.docx]

**S7 Table. The set of raw data for Table 3**

Serum PTH levels (Evocalcet)

| **Dose**  **(μg/kg)** | **Animal No.** | **Before**  **administration** | **1 h** | **4 h** |
| --- | --- | --- | --- | --- |
| 5 | 1-1 | 92.1 | 19.1 | 52.9 |
|  | 1-2 | 97.8 | 25.6 | 115.9 |
|  | 1-3 | 541.4 | 158.9 | 433.3 |
|  | **Mean** | **243.8** | **67.9** | **200.7** |
|  | **S.D.** | **257.8** | **78.9** | **203.9** |
| 150 | 1-4 | 203.0 | 52.9 | 188.4 |
|  | 1-5 | 236.6 | 258.2 | 311.9 |
|  | 1-6 | 57.2 | 36.4 | 81.0 |
|  | **Mean** | **165.6** | **115.9** | **193.8** |
|  | **S.D.** | **95.4** | **123.6** | **115.5** |

Serum PTH levels (Cinacalcet)

| **Dose**  **(μg/kg)** | **Animal No.** | **Before**  **administration** | **1 h** | **4 h** |
| --- | --- | --- | --- | --- |
| 300 | 2-1 | 327.6 | 206.7 | 384.2 |
|  | 2-2 | 84.8 | 42.9 | 103.1 |
|  | 2-3 | 116.8 | 113.4 | 100.2 |
|  | **Mean** | **176.4** | **121.0** | **195.8** |
|  | **S.D.** | **131.9** | **82.2** | **163.1** |
| 500 | 2-4 | 298.9 | 160.9 | 348.3 |
|  | 2-5 | 61.2 | 28.9 | 172.2 |
|  | 2-6 | 370.0 | 66.0 | 370.0 |
|  | **Mean** | **243.4** | **85.3** | **296.8** |
|  | **S.D.** | **161.7** | **68.1** | **108.5** |

Emesis (Evocalcet)

| **Dose**  **(μg/kg)** | **Animal No.** | **Number of vomiting** |
| --- | --- | --- |
| 50 | 1-1 | 0 |
|  | 1-2 | 0 |
|  | 1-3 | 0 |
|  | 1-4 | 0 |
|  | 1-5 | 0 |
|  | 1-6 | 0 |
| 150 | 1-1 | 12 |
|  | 1-2 | 0 |
|  | 1-3 | 0 |
|  | 1-4 | 0 |
|  | 1-5 | 0 |
|  | 1-6 | 0 |

Emesis (Cinacalcet)

| **Dose**  **(μg/kg)** | **Animal No.** | **Number of vomiting** |
| --- | --- | --- |
| 1500 | 2-1 | 0 |
|  | 2-2 | 0 |
|  | 2-3 | 0 |
|  | 2-4 | 0 |
|  | 2-5 | 0 |
|  | 2-6 | 0 |
| 5000 | 2-1 | 6 |
|  | 2-2 | 2 |
|  | 2-3 | 5 |
|  | 2-4 | 1 |
|  | 2-5 | 0 |
|  | 2-6 | 3 |
